# Amyloidogenesis of SARS-CoV-2 Spike Protein

**DOI:** 10.1021/jacs.2c03925

**Published:** 2022-05-17

**Authors:** Sofie Nyström, Per Hammarström

**Affiliations:** Department of Physics, Chemistry and Biology, Linköping University, 58183 Linköping, Sweden

## Abstract

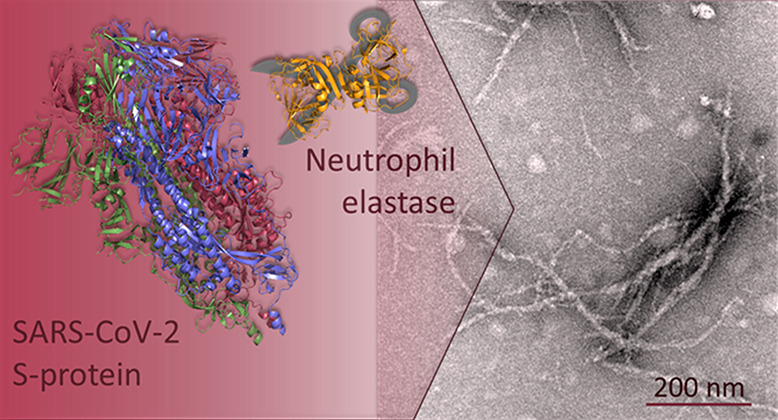

SARS-CoV-2 infection is associated with a surprising number of morbidities. Uncanny similarities with amyloid-disease associated blood coagulation and fibrinolytic disturbances together with neurologic and cardiac problems led us to investigate the amyloidogenicity of the SARS-CoV-2 spike protein (S-protein). Amyloid fibril assays of peptide library mixtures and theoretical predictions identified seven amyloidogenic sequences within the S-protein. All seven peptides in isolation formed aggregates during incubation at 37 °C. Three 20-amino acid long synthetic spike peptides (sequence 192–211, 601–620, 1166–1185) fulfilled three amyloid fibril criteria: nucleation dependent polymerization kinetics by ThT, Congo red positivity, and ultrastructural fibrillar morphology. Full-length folded S-protein did not form amyloid fibrils, but amyloid-like fibrils with evident branching were formed during 24 h of S-protein coincubation with the protease neutrophil elastase (NE) *in vitro*. NE efficiently cleaved S-protein, rendering exposure of amyloidogenic segments and accumulation of the amyloidogenic peptide 194–203, part of the most amyloidogenic synthetic spike peptide. NE is overexpressed at inflamed sites of viral infection. Our data propose a molecular mechanism for potential amyloidogenesis of SARS-CoV-2 S-protein in humans facilitated by endoproteolysis. The prospective of S-protein amyloidogenesis in COVID-19 disease associated pathogenesis can be important in understanding the disease and long COVID-19.

Coronaviruses use the homotrimeric surface spike protein (S-protein) to attach to human cells. Each SARS-CoV-2 S-protein subunit comprises 1273 amino acids.^1^ Four common coronaviruses (OC43, 229E, NL63, and HKU1) infect humans and colonize the respiratory tract. Recently emerged SARS, MERS, and since 2019 also SARS-CoV-2 result in severe disease. Although coronavirus infections are common, not before COVID-19 has such a wide distribution of complex symptoms involving organs other than the respiratory tract been reported. COVID-19 pathogenesis is multifactorial and complex.^2^ Severe COVID-19 includes acute respiratory distress syndrome (ARDS) from innate immune system inflammatory reactions resulting in lung damage;^3^ cytokine storm;^4^ heart damage, including heart muscle inflammation; kidney damage; neurological damage; damage to the circulatory system resulting in poor blood flow. Long-COVID-19 symptoms include persistent emotional illness and mental health conditions resembling neurodegenerative diseases.^2^ What could be the basis for this pathogenesis?

Amyloidosis from several culprit proteins manifests as systemic and localized disorders with many phenotypes overlapping with reported COVID-19 symptoms. It has been proposed that severe inflammatory disease including ARDS in combination with SARS-CoV-2 protein aggregation might induce systemic AA amyloidosis.^5^ Neurotropic colonization and cross-seeding of S-protein amyloid fibrils to induce aggregation of endogenous proteins has been discussed in the context of neurodegeneration.^6^ Notably, blood clotting associated with extracellular amyloidotic fibrillar aggregates in the bloodstream has been reported in COVID-19 patients.^7^ Hypercoagulation/impaired fibrinolysis was demonstrated in blood plasma from healthy donors experimentally spiked with S-protein.^7^

Amyloidosis is associated with cerebral amyloid angiopathy, blood coagulation disruption, fibrinolytic disturbance,^8,9^ FXII Kallikrein/Kinin activation, and thromboinflammation,^10^ suggesting potential links between S-protein amyloidogenesis and COVID-19 phenotypes. We therefore hypothesized a potential molecular link between S-protein and amyloid formation. Inspired by previous hypotheses about human and viral protein amyloids and interactions between them,^11−13^ in particular SARS-CoV spike proteins,^6,14,15^ we asked the question: Is SARS-CoV-2 S-protein amyloidogenic?

We obtained a 316 peptide pool library (divided into two subpools) from a peptide scan through the entire SARS-CoV-2 S-protein (ProteinID: P0DTC2) (Supporting Information). *In vitro* amyloid fibrils were formed in both peptide subpools (Supporting Information, Figure S1). Encouraged by the results, we generated 20-AA peptides from the full-length SARS-CoV-2 S-protein. We aimed to address the most amyloidogenic sequences and used the WALTZ (https://waltz.switchlab.org) prediction algorithm^16^ to identify such segments (Table 1, Supporting Information).

**Table 1:**
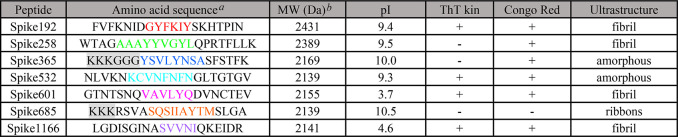
Amino Acid Sequences and Properties of Synthetic SARS-CoV-2 S-Protein peptides

a Residues assigned in color indicate the amyloidogenic segments as predicted by WALTZ. Highlighted in gray are non-native amino acids introduced for solubility.

b Theoretical mass.

Seven amyloidogenic sequences distributed over the entire S-protein were identified and named according to the starting position in the S-protein (Figure S2, Supporting Information). All but one (Spike365) of the predicted sequences are in β-sheet conformation in the SARS-CoV-2 Spike cryo-EM structure in its closed state.^1^ The C-terminal part of the protein (Spike1166) is not resolved in the structure.

Solubilized peptides (0.1 mg/mL, PBS pH 7.5, 10% HFIP) were monitored for *in vitro* amyloid fibril formation kinetics using ThT, Congo red birefringence (CR), and negative stain transmission electron microscopy (TEM).

Fibrils from most of the synthetic peptides were detected within a few hours by at least one assay (Table 1, Figure 1). Spike192, Spike601, and Spike1166 fulfilled all our amyloid criteria: sigmoidal ThT kinetics, Congophilicity, and fibrillar ultrastructure (Figure 1, Table 1). Spike192 formed exceptionally well-ordered fibrils comparable to a mix of all peptides (Figures 1C and S3C).

**Figure 1:**
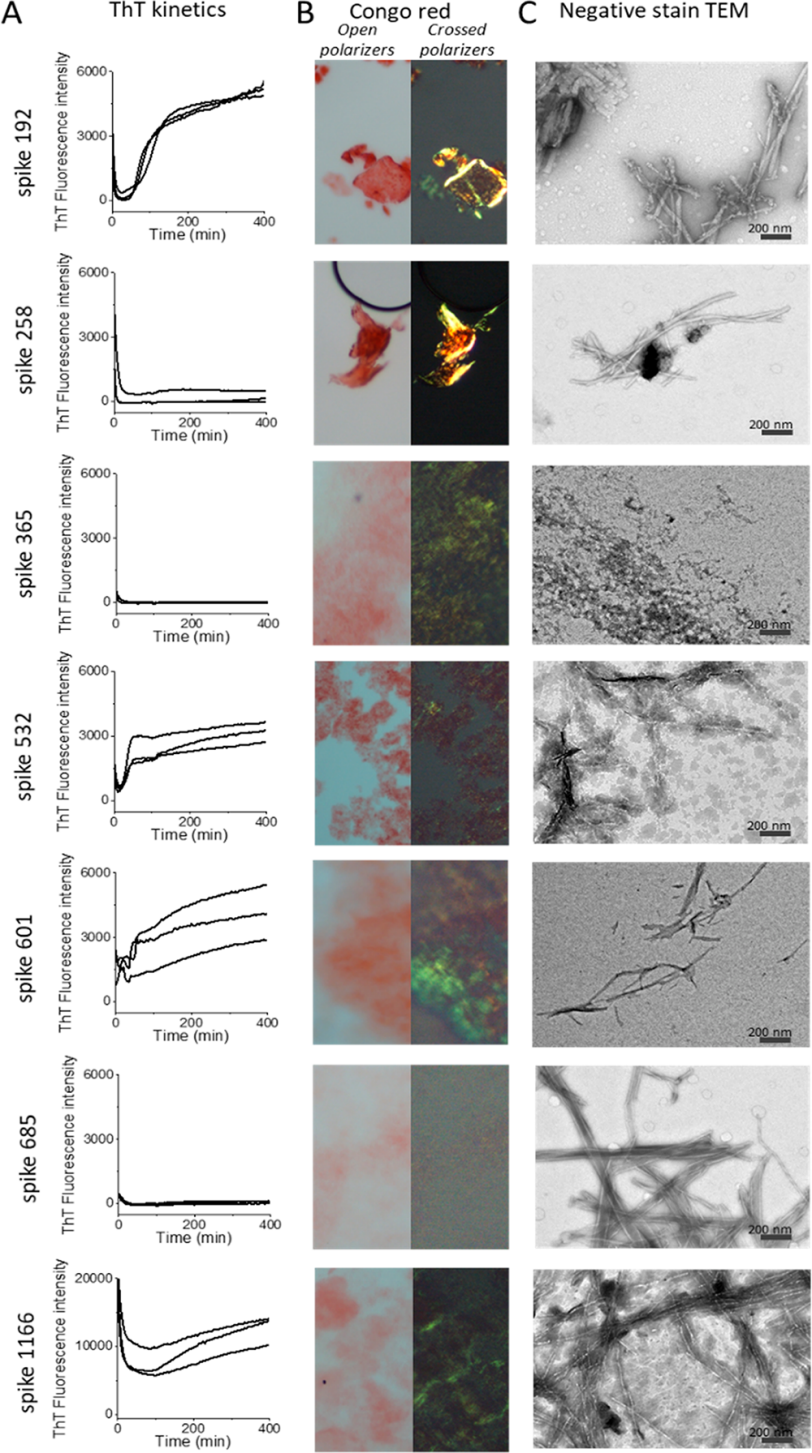
Amyloid fibril assays of SARS-CoV-2 S peptides (0.1 mg/mL). (A) ThT fluorescence fibril formation kinetics. (B) Congo red birefringence microscopy. (C) Negative stain TEM ultrastructure.

What would be a plausible mechanism for S-protein fibril formation during SARS-CoV-2 infection? SARS-CoV-2 S-protein is fairly stable (*T*_m_ > 50 °C)^17^ and would not readily denature spontaneously. Furthermore, such a large protein with complex folding will not easily misfold into an amyloid state. However, proteolysis is an obvious candidate mechanism.

Endoproteolysis of precursor proteins is a well-known molecular initiation mechanism in several amyloidoses, notably Alzheimer′s disease (AβPP), British and Danish dementia (ABri/ADan), and Finnish familial amyloidosis (AGel). Proteolysis of full-length proteins is also evident in many other amyloid disease deposits from ATTR, ALys, AA, and ASem1.^18,19^

SARS-CoV-2 S-protein is proteolyzed during infection by host furin-like enzymes and by serine proteases such as the transmembrane protease, serine 2 (TMPRSS2), at the cell surface^20^ and is further proteolyzed during inflammation.

Neutrophils are the dominating class of leukocytes and a first responder during acute inflammation. Neutrophils are recruited to the bronchoalveolar space of patients infected with a range of different respiratory viruses, including SARS-CoV-2.^21^ Neutrophils act by phagocytosis of opsonized pathogens and by extracellular release of enzymes such as neutrophil elastase (NE). NE is a serine protease coupled to obstructive lung diseases such as cystic fibrosis, chronic obstructive pulmonary disease,^22^ and alpha-1-antitrypsin deficiency.^23^

The amino acid sequence of SARS-CoV-2 S-protein was subjected to *in silico* proteolytic cleavage by NE using Expasy Peptide cutter. One of the resulting peptides, Spike194–213, closely matched Spike192, only frame shifted by two amino acids (Supporting Information), implying a testable hypothesis.

We subjected full-length SARS-CoV-2 S-protein to NE cleavage *in vitro*. S-protein showed a complex thermal unfolding trajectory with multiple transitions around 45–65 °C and a major unfolding transition with a midpoint of denaturation (*T*_m_) of 79 °C (Figures 2A and S4A) with differential scanning fluorimetry (DSF) (Figures 2 and S4), similar to literature values for full-length S-proteins,^24^ confirming folded protein at starting point. S-protein refolded upon cooling albeit noncooperatively (Figure 2A). NE unfolded irreversibly (*T*_m_ 59 °C) (Figure 2B). Co-incubated S-protein+NE only showed an obvious transition for NE and did not refold upon cooling, suggesting that S-protein had been cleaved by NE (Figure 2C). Mass spectrometry verified digestion since only the S-protein+NE experiment revealed peptide peaks (Figure 2D–F).

**Figure 2:**
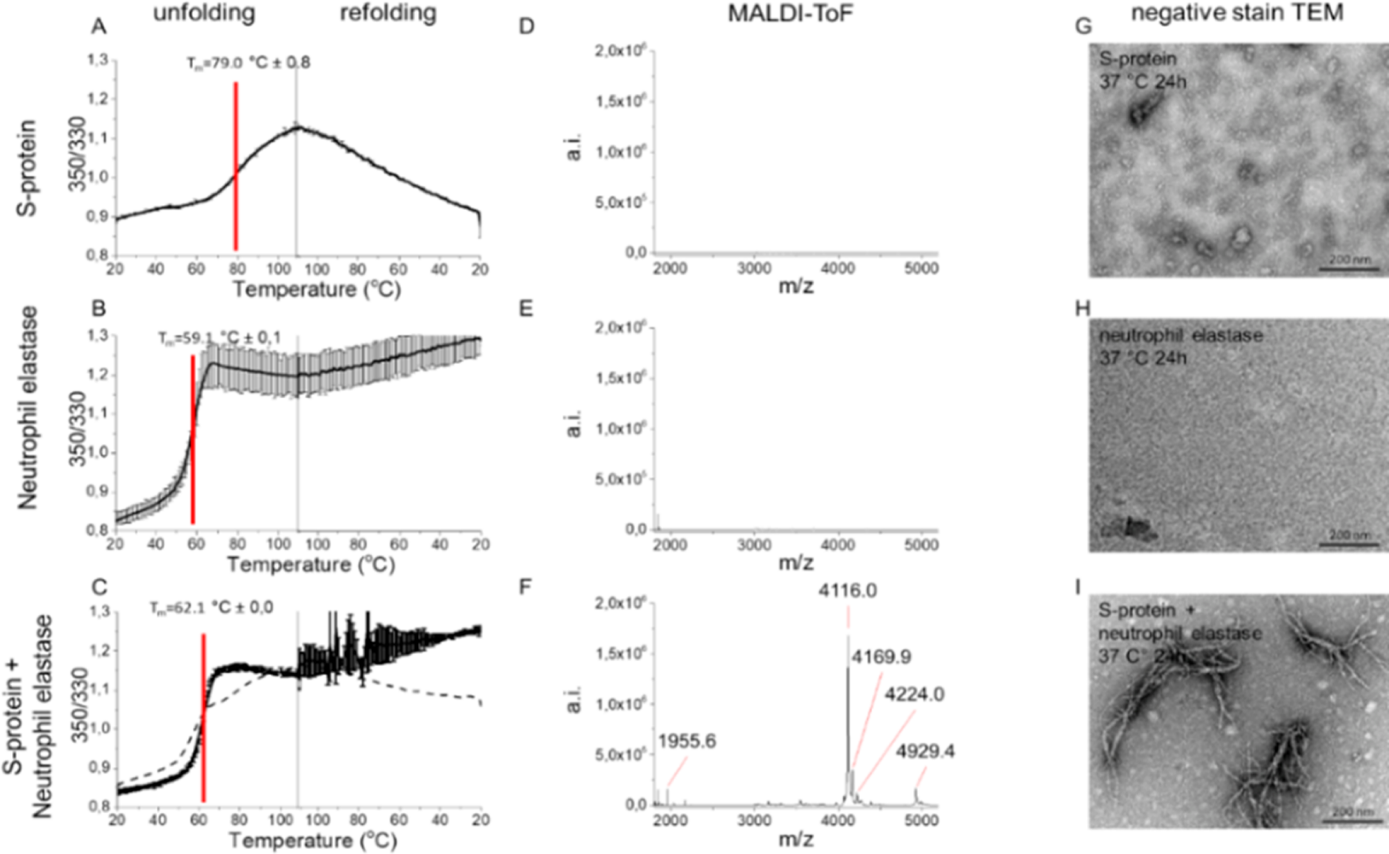
S-protein proteolysis by NE renders amyloid-like fibrils. Thermostability of (A) SARS-CoV-2 S-protein, (B) NE, (C) S-protein+NE, measured by DSF. Dashed line in (C) is the mathematical sum of S-protein and NE, respectively, from (A) and (B) supporting cleavage of S-protein by NE. MALDI-ToF spectra of C18 isolated peptides of (D) S-protein, (E) NE, and (F) S-protein+NE (6 h, 37 °C). TEM micrographs of (G) S-protein alone depicting the expected trimers, (H) NE alone, and (I) S-protein+NE coincubated at pH 8.4, 24 h, 37 °C.

Most importantly, we discovered amyloid-like fibril formation upon proteolytic cleavage using TEM. Neither NE nor SARS-CoV-2 S-protein incubated alone formed fibrils (Figure 2G–H). Fibrils were found only after co-incubation of the two proteins (Figure 2I). The fibrils showed unusual morphology with evident branching (Figure 2I), suggesting involvement of proteolytically nicked S-protein within the fibril, rendering nodes for branching of different amyloidogenic sequences (Figures 2I and S5).

We then performed LC-MS/MS analysis of peptides formed after 1 min and 6 h of digestion at 2:1 excess of NE over S-protein. We identified 98 NE cleavage peptides (Table S1) from the S-protein structure and classified these into three groups: (i) formed after 1 min (Figure 3A, B), (ii) formed after 1 min and still present after 6 h (Figure 3A, C), and (iii) only present after 6 h (Figure 3A, D). Initial cleavage and further digestion (group i) occurred mainly within the S2 domain with abundant cleavage of the HR domains and in the C-terminal part of S1. NTD and RBD were much less affected (Figure 3A). Three persistent peptides formed after initial cleavage (group ii) originated from NTD and RBD. Several peptides only formed after 6 h of incubation (group iii). Strikingly, the peptide from segment 194–203 (FKNIDGYFKI, included in Spike192) was part of this group and was highly abundant after 6 h (Table S2). Three peptides containing segments from our seven spike peptides (Figure 3A, E) were formed as free peptides (Spike192, Spike258, and Spike1166) still present after 6 h of co-incubation, two were digested early and disappeared (Spike532 and Spike685), and two were likely still present in the parent nicked S-protein (Spike365 and Spike601). Hence, the observed formed branched fibrils (Figures 2I and S5) are likely composed of a mix of fibrils initiated by an amyloidogenic peptide seed recruiting nicked S-protein for elongation and branching.

**Figure 3:**
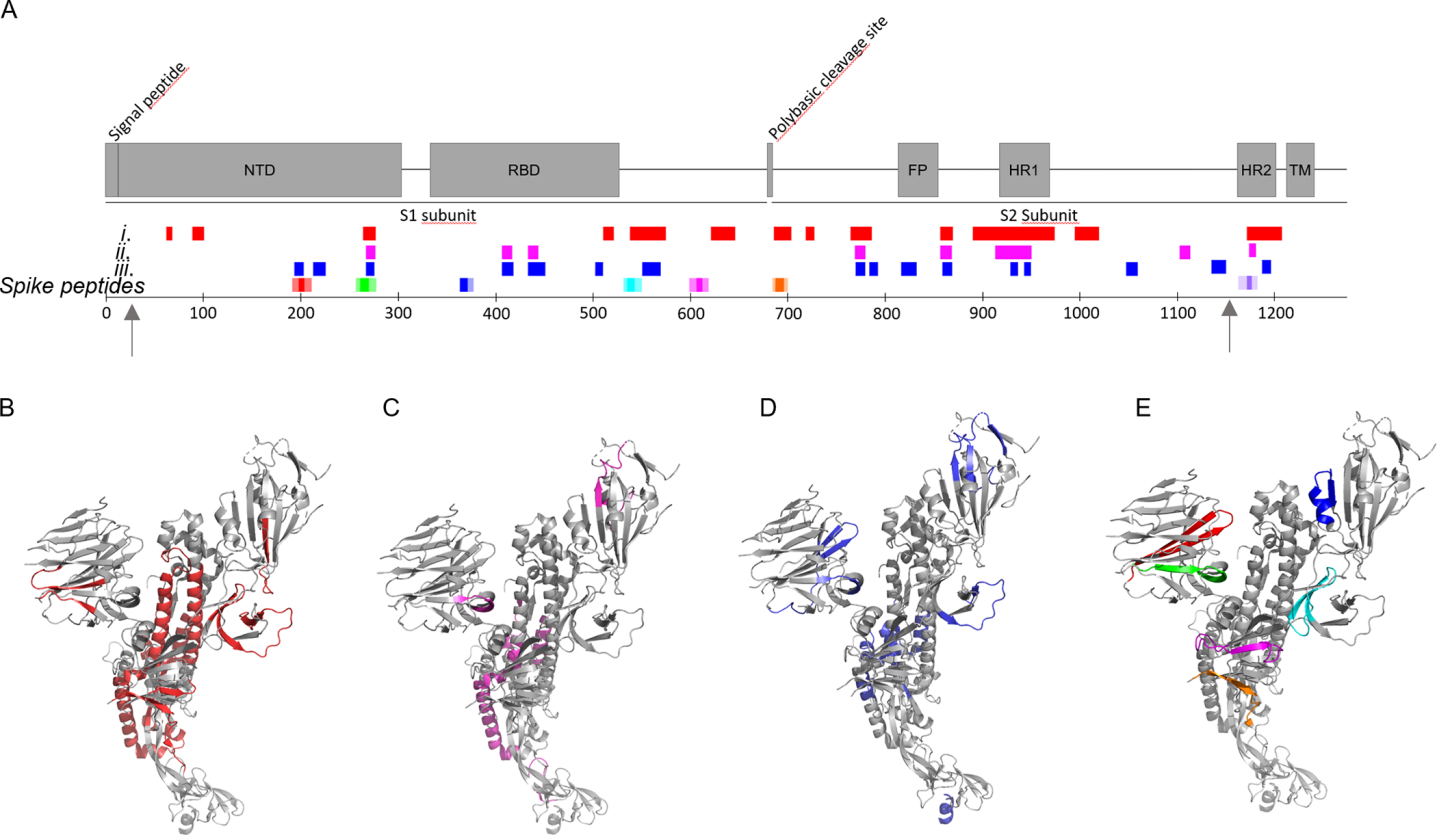
NE cleavage sites within full-length S-protein. Arrows indicate limits of the cryo-EM structure 27–1146. (A) Peptides identified by LC-MS/MS (Table S1) in correlation to the amyloidogenic Spike peptides (cf. Figure 1 and Table 1) and the S-protein domain structure: after (i) 1 min (red), (ii) 1 min and still persistent at 6 h (magenta), (iii) only present after 6 h incubation (blue). (B–E) Same color code for cleaved peptide groups (i)–(iii) and spike peptides mapped onto the protomer cryo-EM S-protein structure PDB 6VXX.^1^

We performed fibrillation experiments identical to that for the other spike peptides also on the short fragment 194–203 (Figure 4A, B). The peptide was less amyloidogenic than Spike192 (1 mg/mL was required to form fibrils compared to 0.1 mg/mL for Spike192) (Figure 4B). Formed fibrils of Spike194–203 were however amyloidogenic by our three criteria, ThT kinetics, Congo red birefringence, and fibrillar ultrastructure (Figure 4A, B). It is worth noting that the Spike194–203 peptide lacks one amino acid in the predicted amyloidogenic sequence. To test the significance of this amino acid deletion in the peptide, we performed a simple *in silico* mutation experiment where we substituted the final tyrosine in the amyloidogenic segment of Spike192 with a glycine to mimic its deletion. The *in silico* substitution Y204G abolished the amyloid prediction (Figure S6), demonstrating that removal of this amino acid will alter the amyloidogenic properties of the peptide. Fibril “shaving” is known for several amyloidogenic proteins and peptides.^25^ It is possible that Y204 is cleaved after S-protein aggregation.

**Figure 4:**
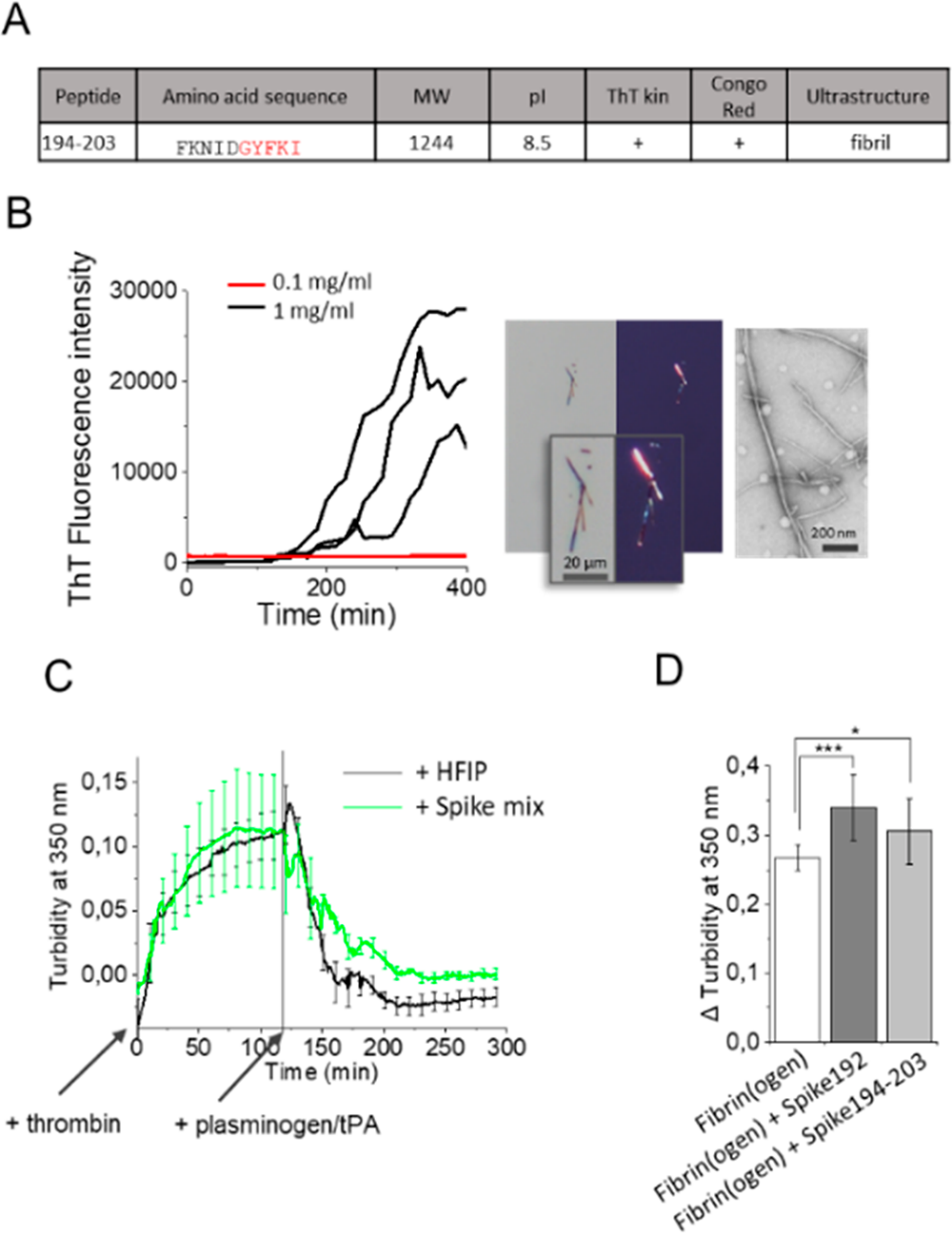
(A) Spike194–203 properties. (B) Spike194–203 amyloidogenicity monitored by ThT kinetics, Congo red birefringence, and TEM. (C) Thrombin activated fibrin formation and plasminogen/tPA-induced fibrinolysis in the absence and presence of seven-peptide-mix amyloid fibrils (Table 1, Figure S3). (D) Turbidity difference after fibrin formation and fibrinolysis in the absence and presence of Spike192 fibrils and Spike194–203 fibrils (*n* = 10–12 replicates of each reaction, corrected for background scatter).

It is known that S-protein affects the formation of persistent amyloid-like microclots in human blood, a potential pathological cause of long COVID-19 symptoms.^26^ We performed a thrombin induced fibrinogen to fibrin conversion followed by plasminogen tPA assay^27^ in the presence and absence of Spike peptide fibrils (Supporting Information).

The addition of 10 μg/mL amyloid fibrils formed from a mix of the seven spike peptides (Table 1, Figure S3) during fibrin formation decreased the fibrinolysis (Figure 4C). Furthermore, the addition of 2% fibrils (from 1 mg/mL stock, total 20 μg/mL) of Spike192 and 194–203 increased persistent plasmin indigestible fibrin (Figure 4 D). As expected, the more amyloidogenic Spike192 induced more plasmin resistant fibrin clots than did Spike194–203. Our reductionist assay appears to replicate results from human plasma samples.^7^

We tested two fluorescent analogues of positron emission tomography (PET) amyloid tracers, CN-PiB (benzothiazole analogue of Pittsburgh compound B) and DF-9 (stilbene analogue of Florbetaben), known to bind to neurological Aβ amyloid and cardiac AL, AA, and ATTR amyloid and found strong binding with concomitant fluorescence response toward Spike192 fibrils *in vitro* (Figure S7). As a translational strategy, PET imaging may hence serve as an option for clinical studies to complement liquid biopsies to assess amyloid microclots.^26^

In conclusion, we herein proposed a simple molecular mechanism for how SARS-CoV-2 S-protein endoproteolyzed by NE can form amyloidogenic S-peptides, such as segment 194–203, and lead to exposure of multiple amyloidogenic segments in proteolytically nicked S-protein.

It is possible that other amyloidogenic peptides and S-protein nicked by other proteases could be involved if this process occurs *in vivo*. We found that all common coronaviruses infecting humans contain amyloidogenic sequences (Figure S8A). Nonetheless, the magnitude of diverse COVID-19 symptoms was not previously reported. The segment 194–213 is unique for SARS-CoV-2 (Figure S8B) which, in combination with acute inflammation and neutrophil recruitment known to be more prevalent in COVID-19 compared to other viral infections, could explain the putative COVID-19 associated amyloid formation. It should be mentioned that amyloidosis is rather common in the elderly population^18^ and its associations with viral infections is a matter of discussion.^13^ Recent studies demonstrate that COVID-19 recovered patients have an increased risk of type II diabetes, an amyloid associated disease.^28,29^ While our study is limited to *in vitro* findings of pure preparations of peptides and proteins, the results propose taking S-protein amyloidogenesis into account when studying COVID-19 and long COVID-19 symptoms.

## Supporting Information Available

Experimental procedures; bulk S-protein peptide library sequences; selection of amyloidogenic segments for peptide synthesis and comparison with peptides rendered by *in silico* elastase digestion (PDF)
Elastase peptides at 1 min vs 6 h (XLSX)
Quantification of abundant peaks (XLSX)

## Abbreviations

CNS: central nervous system
SARS-CoV-2: Severe Acute Respiratory Syndrome Coronavirus-2
S-protein: spike protein
ThT: thioflavin T
CN-PiB: Cyano-Pittsburgh compound B
TEM: transmission electron microscopy
DSF: differential scanning fluorimetry
