# Supplementary material for: Amyloidogenesis of SARS-CoV-2 Spike Protein
Source: J Am Chem Soc. 2022 May 17;144(20):8945–50. doi: 10.1021/jacs.2c03925 (PMC9136918; doi:10.1021/jacs.2c03925)
Supplement: Supplementary file 1 — ja2c03925_si_001.pdf [file ja2c03925_si_001.pdf]

Supporting information

Amyloidogenesis of SARS-CoV-2 Spike Protein

Sofie Nyström*‡, Per Hammarström*‡

‡Department of Physics, Chemistry and Biology, Linköping University, 58183, Linköping, Sweden.

*e-mail: sofie.nystrom@liu.se; per.hammarstrom@liu.se

Table of contents

Supplementary Methods

Supporting information: Bulk S-protein peptide library sequences

Supporting information: Selection of amyloidogenic segments for peptide synthesis and comparison with peptides rendered by *in silico* elastase digestion

Figure S1: Fibrils formed from bulk S-protein peptide library

Figure S2: Structural context of the Spike peptides

Figure S3: Fibrillation of mix of the seven Spike peptides

Figure S4: Thermal denaturation by DSF

Figure S5: Raw transmission electron micrographs of full-length SARS-CoV-2 S-protein, Neutrophil Elastase and S-protein + Neutrophil Elastase incubated at 37 °C for 24h

Figure S6: *In silico* mutation of one amino acid in the amyloidogenic segment in Spike192

Figure S7: Fluorescence micrographs and spectra of Spike192 fibrils stained with fluorescent analogs of amyloid PET tracers

Figure S8: WALTZ amyloidogenic sequence predictions of S-protein sequences from seven corona viruses known to infect humans

Table S1: S-protein peptides formed by elastase clevage 1 min vs 6 h by LC-MS/MS (separate excel file)

Table S2: Quantification of abundant S-peptide peaks by LC-MS/MS (separate excel file)

Supporting Methods

Solubilization and fibrillation of bulk peptide library

A bulk library of 15-mer peptides with 11 amino acid overlap was purchased from GenScript, Netherlands (cat no catalog no RP30020), (Supporting information). The peptides were delivered as lyophilized powder in two subpools. Each subpool was treated separately as follows.

The lyophilized powder was reconstituted in autoclaved, ice-cold PBS (0.14 M NaCl, 0.0027 M KCl, 0.01 M PO_4^{3-} (Medicago)) to a total concentration of 2 mg/ml peptide mixture (~12 µg/ml per peptide) and subsequently sonicated in water bath for 15 minutes. The resulting turbid mixture was transferred to centrifugation microtube and spun at 20 000 g at 4 °C for 20 minutes. The supernatant (hereafter called soluble fraction) was transferred to a fresh tube and the pellet was supplemented with hexafluoroisopropanol (HFIP) at a volume corresponding to 10 % of the initially added PBS (Hereafter called insoluble fraction). This resulted in a clear solution.

The soluble fraction was supplemented with 10% HFIP to match HFIP concentration on the insoluble fraction. PBS was added to the insoluble fraction to reach a final concentration of 10% HFIP. 400 µl of each of the four preparations were aliquoted to tightly sealed glass vials and placed at 37 °C for 24 hours.

Selection of amyloid prone segments

Custom made peptides were selected from the full-length SARS-CoV-2 spike protein (protein ID: P0DTC2) with the aim to generate amyloid prone peptides. The primary sequence of the protein was subjected to the WALTZ algorithm (https://waltz.switchlab.org/) [1]. The algorithm was set to High selectivity to avoid false positives. The algorithm predicted 7 amyloidogenic sequences comprising 5-9 amino acids respectively (supporting information). The WALTZ predicted sequence was placed in the middle and flanking amino acids were selected to include hydrophobic and exclude charged residues. The peptides were named based on the number of the first amino acid in the respective sequence. Based on this information we selected peptides stretching over 20 amino acids. Due to solubility issues for some peptides, charged residues were reincluded and in some instances (Spike365 and Spike685) additional lysines and glycines were included to generate producible peptides. Spike258 was produced with 21 amino acids to allow for both the N-terminal tryptophan and the C-terminal lysine to be included.

AlphaFold2[2] was performed on the selected peptides to predict structure as free peptides. Three peptides: Spike258, Spike365 and Spike685, that are in β-strand conformation in the full-length protein are in helical conformation as free peptides (Figure S2B). One of the peptides (Spike601) was predicted by Alphafold2 to be random coil and Spike1166 was predicted to be an α-helix. Spike365 and Spike685 have been N-terminally modified with solubilizing sequences to enable production. Alphafold2 was performed on the sequences in Table 1.

Solubilization and fibrillation of custom-made peptides

Custom-made peptides (main text Table 1) were delivered from GenScript, Netherlands as lyophilized powder. 100% HFIP was added to each peptide to reach a final peptide concentration of 10 mg/ml. The vials were sonicated in water bath for 15 minutes and subsequently frozen at -20 °C until needed.

On day of fibrillation, the solubilized peptides were thawed at room temperature and diluted in HFIP to reach a peptide concentration of 1 mg/ml and sonicated in water bath 15 minutes. Triplicate samples for each peptide were prepared by adding HFIP solubilized peptide to ice cold phosphate buffered saline (PBS) pH 7.4, supplemented with ThT to reach a final concentration of 0.1 mg/ml

peptide, 2 µM ThT and 10% HFIP. MALDI-ToF mass spectra were collected on the dissolved peptides to verify purity (Figure S2C) Peptides at a concentration of 0.1 mg/ml dissolved in PBS was mixed 50/50 with alpha-Cyano-4-hydroxycinnamic acid matrix and were analyzed by UltrafleXtreme MALDI system (Bruker Daltonics, Bremen, Germany).

A mixture of all peptides was also prepared by mixing equal volumes of each peptide preparation. This renders a total peptide concentration of 0.1 mg/ml and a concentration of 0.014 mg/ml of each constituting peptide. The samples were distributed in 96-well black untreated half area plates with transparent bottom (Corning costar 3880) placed on a bed of ice. The plate was firmly sealed with aluminum and plastic sealing film to prevent evaporation of HFIP. The plate was placed at 37 °C in a Tecan Infinite M1000 Pro plate reader with linear shaking between measurements with amplitude 2 mm and frequency 654 rpm. ThT intensity was monitored by bottom read mode with excitation at 440 nm and emission at 500 nm every 5 minutes for 24 hours. The mixture of peptides resulted in amyloid fibril formation with sigmoidal ThT kinetics suggesting a nucleation dependent mechanism (Figure S3A). The fibril morphology from the mixture (Figure S3B) most closely resembled that of fibrils composed of the well-ordered Spike192 (cf. Figure S3B; Figure S3C), suggesting that this peptide is dominating the fibril structures in the mixture.

Corresponding samples but with ThT omitted was also included on the plate for upstream experiments (below referred to as unstained reactions).

Transmission electron microscopy

TEM grids from each of the samples were prepared from the unstained fibrillation reactions as follows: Five µL of samples were placed on carbon coated copper grids (Carbon-B, Ted Pella Inc.) and were incubated for 2 minutes. Excessive salt was removed with one wash of 5 µL deionized water and grids were negatively stained with 2% uranyl acetate for 30 s. Grids were blotted dry and air dried overnight.

Transmission electron microscopy (TEM) imaging was performed using a Jeol JEM1400 Flash TEM microscope operating at 80 kV (Main text Figure 1, 2, 4, Figure S1, Figure S3, and Figure S5).

Congo red birefringence

Aliquots from the unstained fibrillation reactions were prepared for evaluation of Congo red birefringence as follows: Five µl of Congo red stock solution (100 µM in milliQ water) was added to 45 µl of 0.1 mg/ml Spike-peptide fibrils in solution resulting in a molar ratio of Spike-peptide:dye 4:1. Stained fibrils were left to self-sediment over-night at 20 °C. 5 µl from the bottom of the pelleted samples were transferred to superfrost gold glass slides (Thermo Fisher, Walldorf, Germany) and allowed to dry. The dried droplets were covered with fluorescence mounting medium (Dako, Glosrup, Denmark). Congo red stained samples were analyzed using a Nikon light microscope equipped with polarizers for both incoming light and in front of the detector (Main text Figure 1 and 5).

Elastase digestion of full-length S-protein

Full-length wild-type SARS-CoV-2 S-protein (protein ID: P0DTC2) expressed in human HEK293 cells (Sigma-Aldrich catalogue # AGX819) was subjected to proteolytic cleavage by Human elastase expressed in human leucocytes (Sigma-Aldrich, catalogue # 324681) as follows:

The S-protein was delivered in Tris buffer pH 8 at a concentration of 5.8 µM (on a monomer basis). Elastase was delivered as lyophilized powder and was reconstituted in 10 mM Tris-HCl pH 8.4 (Tris-buffer) to a final concentration of 13.5 µM. Three sample types were prepared; 1 Spike diluted 1:1 in Tris-buffer; 2 Elastase diluted 1:1 in Tris buffer; 3 Spike and Elastase stocks mixed 1:1. All samples

were incubated at 37 °C for 24 hours. After 24 hours PMSF was added to all samples to a final concentration of 1 mM. TEM grids were prepared, and TEM analysis was performed as described above.

Thermal stability of elastase digested S-protein

Thermal unfolding and refolding of SARS-CoV-2 S-protein, monitored by spectral shift due to tryptophan exposure, in the presence or absence of elastase was assayed to verify folded protein from initiation and lack of refolding if nicking by protelolysis of S-protein was occurring. Samples were prepared with recombinant full-length SARS-CoV-2 S-protein 1 µM on a monomer basis (S-protein contains 12 Trp per monomer) in the presence or absence of neutrophil elastase 2.5 µM (3 Trp). Elastase alone was also assayed for comparison. All samples were prepared in 10 mM Tris-HCl buffer pH 8.4. Thermal stability scan was initiated 15 min after mixing of samples at 20 °C. Analysis was performed by Trp fluorescence by nanoscale differential fluorimetry (350nm/330 nm emission ratio) using the Prometehus NT-48 instrumentation (Nanotemper). Settings: Thermal scan, 1 °C/min, 20-110 °C for unfolding and followed by refolding at 1 °C/min (Main text Figure 2, Figure S4).

Mass spectrometry of elastase digested S-protein

Samples from the same reactions as for DSF were incubated in parallel at 37 °C for 6 h prior to preparation of samples for mass spectrometry. The reaction was stopped by mixing with equal volumes of 6 M GuHCl, 0.2 % TFA to a final concentration of 3 M GuHCl and 0.1% TFA. Samples were Zip-tipped using C4 and C18 Zip-tips respectively as described by the manufacturer (Millipore). Peptides isolated by C18 were mixed 50/50 with alpha-Cyano-4-hydroxycinnamic acid matrix and protein samples zip tipped with C4 were mixed with sinapinic acid as matrix for MALDI-ToF. Protein mass spectra were acquired on an UltrafleXtreme MALDI-TOF mass spectrometer (Bruker Daltonics Bremen Germany) instrument operated in the linear positive ion mode with flexControl software (Version 3.4, Bruker Daltonics). The MS spectra obtained were analyzed using flexAnalysis software (Version 3.4, Bruker Daltonics) (Main text Figure 2). No peaks were recovered above 50000 Da from the acquired spectra even for C4 isolated proteins. Elastase, where added, showed a peak with a centroid at 27100 ± 3 Da.

LC–MS/MS and peptide identification

The S-protein at a final concentration of 1.45 µM (on a monomer basis) was co-incubated with elastase at a final concentration of 2.7 µM in 10 mM Tris-HCl pH 8.4 (Tris-buffer) for 1 min or 6 h at 37 °C. Undigested S-protein was run as a control to check for possible *in vitro* degradation peptides. The reaction was stopped by mixing with equal volumes of 6 M GuHCl, 0.2 % TFA to a final concentration of 3 M GuHCl and 0.1% TFA. Samples were desalted using Ziptips C18 as described by the manufacturer (Millipore). Samples were eluted using 50% and 70% acetonitrile with 0.1 % TFA and vacuum dried using Speedvac concentrator (Thermo Scientific Savant).

The samples were redissolved in 0.1% formic acid in milli-Q water and were applied to an EASY-nanoLC II system (Thermo Fisher Scientific) with a C18 reverse chromatography column 20 mm × 100 µm C18 pre column followed by a 100 mm × 75 µm C18 column with particle size 5 µm (NanoSeparations, Nieuwkoop, Netherlands) and peptides were separated at a flow rate of 300 nL/min by a gradient of 0.1% formic acid in water (A) and 0.1% formic acid in acetonitrile (B) as follows: from 2% B to 30% B in 60 minutes; from 30% B to 100% B in 60 minutes.

Automated online analyses were performed in positive mode by LTQ Orbitrap Velos Pro hybrid mass spectrometer (Thermo Scientific) equipped with a nano-electrospray source with Xcalibur software (v.2.6, Thermo Scientific). Full MS scans were collected with a range of 350–1800 *m/z*, a resolution of 30 000 (*m/z* 200), the top 20 most intense multiple charged ions were selected with an isolation window

of 2.0 and fragmented in the linear ion trap by collision-induced dissociation with normalized collision energy of 35%. Dynamic exclusion was enabled ensuring peaks selected for fragmentation were excluded for 60 s.

Peptides were identified using using Sequest HT in Proteome Discoverer (Thermo Fisher Scientific, San Jose, CS version 2.5.0.400) and the cRAP database (cRAP protein sequences (thegpm.org)) merged with - P0DTC2 sequence for the SARS-CoV-2 S-protein (UniProtKB). and elastase cleavage prediction for peptide identification allowing missed cleavage sites. The following search parameters were used: elastase as a digestion enzyme; maximum number of missed cleavages 8; fragment ion mass tolerance 0.10 Da; parent ion mass tolerance 15.0 ppm.

SARS-CoV-2 S-protein peptides were quantified using two methods: peak intensity and spectral counts. Based on this, abundances of peptides originating from SARS-CoV-2 S-protein were compared for the 3 S-protein samples: undigested, incubated for 1 min with elastase, incubated for 6 h with elastase (Table S1 and Table S2).

Fibrin formation and fibrinolysis

Formation of fibrin from fibrinogen and subsequent fibrinolysis was monitored in Tecan Infinite M1000 Pro plate reader in Corning Costar non-binding half area plates 3881 by measuring turbidity increase and decrease with absorbance at 350 nm. The experiments were performed at 37 °C [3].

Human fibrinogen (plasminogen free) purified from human plasma (Merck) at a final concentration of 0,5 mg/ml in fibrin buffer (20 mM Hepes pH 7.4, 120 mM NaCl, 1 mM $CaCl_2$) was used in 200 µl per well. Amyloids formed from the mix of seven spike peptides was added to the fibrinogen to a final concentration of 10 µg/ml and buffer with corresponding volume of HFIP as the fibril preparations as result of solubilization of the peptides was used as control. Fibrin formation was induced by activation with 0,1 U/µl Thrombin (Sigma). When equilibrium was reached after 120 minutes, the plate was ejected from the plate reader. Fibrin clots were observed in all wells where both fibrinogen and thrombin had been added. Fibrinolysis was induced by adding plasminogen from human serum (Sigma) and its activator human tissue plasminogen activator (tPA) (Sigma) at a final concentration of 0.5 µM and 1.2 nM respectively. The fibrinolysis was again monitored by A350 to detect decrease in turbidity as the fibrin clot was digested by plasmin (Main text Figure 4C).

To explore the effect of individual spike peptide amyloid fibrils, the experiment was repeated but using spike amyloid generated from pure preparations of spike192 and spike194-203 respectively. Spike amyloid was added to the reaction at a final concentration of 20 µg/ml and 150 µl total volume was used in each well. For each individual reaction (10-12 replicates), the average of three sample specific control samples with fibrinogen and the respective spike amyloid or HFIP control, but without thrombin, were subtracted. The difference in turbidity between point of fibrin formation equilibrium at 120 minutes and fibrinolysis equilibrium at 270 minutes were calculated (Main text Figure 4D).

Staining with fluorescent PET amyloid ligand analogues CN-PiB and DF-9

Unstained fibrils of Spike192 formed at 1 mg/ml was diluted to 0.1 mg/ml in PBS buffer pH 7.4 and were stained with a final concentration of 1 µM CN-PiB or DF-9 over night. 3 µl from the bottom of the sedimented samples were transferred to superfrost gold glass slides (Thermo Fisher, Walldorf, Germany) and allowed to dry. The dried droplets were covered with fluorescence mounting medium (Dako, Glosrup, Denmark). Stained samples were analyzed using a Leica600DM epifluorescence microscope equipped with long band pass filters and a hyperspectral camera (SpectraCube, ASI, Israel). Settings: excitation 350 nm emission 400-700 nm, 25 ms exposure, 20x objective. (Figure S7).

Supporting information: Bulk S-protein peptide library sequences

Marked in yellow are peptides marked as amyloidogenic based on WALTZ “best overall performance” settings [1].

Red letters were indicated as amyloidogenic with WALTZ “high specificity” settings.

Subpool 1

SARS-CoV-2 Spike_1	MFVFLVLLPLVSSQC
SARS-CoV-2 Spike_2	LVLLPLVSSQCVNLT
SARS-CoV-2 Spike_3	PLVSSQCVNLTTRTQ
SARS-CoV-2 Spike_4	SQCVNLTTRTQLPPA
SARS-CoV-2 Spike_5	NLTTRTQLPPAYTNS
SARS-CoV-2 Spike_6	RTQLPPAYTNSFTRG
SARS-CoV-2 Spike_7	PPAYTNSFTRGVYYP
SARS-CoV-2 Spike_8	TNSFTRGVYYPDKVF
SARS-CoV-2 Spike_9	TRGVYYPDKVFRSSV
SARS-CoV-2 Spike_10	YYPDKVFRSSVLHST
SARS-CoV-2 Spike_11	KVFRSSVLHSTQDLF
SARS-CoV-2 Spike_12	SSVLHSTQDLFLPFF
SARS-CoV-2 Spike_13	HSTQDLFLPFFSNVT
SARS-CoV-2 Spike_14	DLFLPFFSNVTWFHA
SARS-CoV-2 Spike_15	PFFSNVTWFHAIHVS
SARS-CoV-2 Spike_16	NVTWFHAIHVSGTNG
SARS-CoV-2 Spike_17	FHAIHVSGTNGTKRF
SARS-CoV-2 Spike_18	HVSGTNGTKRFDNPV
SARS-CoV-2 Spike_19	TNGTKRFDNPVLPFN
SARS-CoV-2 Spike_20	KRFDNPVLPFNDGVY
SARS-CoV-2 Spike_21	NPVLPFNDGVYFAST
SARS-CoV-2 Spike_22	PFNDGVYFASTEKSN
SARS-CoV-2 Spike_23	GVYFASTEKSNIIRG
SARS-CoV-2 Spike_24	ASTEKSNIIRGWIFG
SARS-CoV-2 Spike_25	KSNIIRGWIFGTTLD
SARS-CoV-2 Spike_26	IRGWIFGTTLDSKTQ
SARS-CoV-2 Spike_27	IFGTTLDSKTQSLLI
SARS-CoV-2 Spike_28	TLDSKTQSLLIVNNA
SARS-CoV-2 Spike_29	KTQSLLIVNNATNVV
SARS-CoV-2 Spike_30	LLIVNNATNVVIKVC
SARS-CoV-2 Spike_31	NNATNVVIKVCEFQF
SARS-CoV-2 Spike_32	NVVIKVCEFQFCNDP
SARS-CoV-2 Spike_33	KVCEFQFCNDPFLGV
SARS-CoV-2 Spike_34	FQFCNDPFLGVYYHK
SARS-CoV-2 Spike_35	NDPFLGVYYHKNNKS
SARS-CoV-2 Spike_36	LGVYYHKNNKSWMES
SARS-CoV-2 Spike_37	YHKNNKSWMESEFRV
SARS-CoV-2 Spike_38	NKSWMESEFRVYSSA
SARS-CoV-2 Spike_39	MESEFRVYSSANNCT
SARS-CoV-2 Spike_40	FRVYSSANNCTFEYV
SARS-CoV-2 Spike_41	SSANNCTFEYVSQPF
SARS-CoV-2 Spike_42	NCTFEYVSQPFLMDL
SARS-CoV-2 Spike_43	EYVSQPFLMDLEGKQ
SARS-CoV-2 Spike_44	QPFLMDLEGKQGNFK
SARS-CoV-2 Spike_45	MDLEGKQGNFKNLRE
SARS-CoV-2 Spike_46	GKQGNFKNLREFVFK
SARS-CoV-2 Spike_47	NFKNLREFVFKNIDG
SARS-CoV-2 Spike_48	LREFVFKNIDGYFKI
SARS-CoV-2 Spike_49	VFKNIDGYFKIYSKH
SARS-CoV-2 Spike_50	IDGYFKIYSKHTPIN
SARS-CoV-2 Spike_51	FKIYSKHTPINLVRD
SARS-CoV-2 Spike_52	SKHTPINLVRDLPQG
SARS-CoV-2 Spike_53	PINLVRDLPQGFSAL
SARS-CoV-2 Spike_54	VRDLPQGFSALEPLV
SARS-CoV-2 Spike_55	PQGFSALEPLVDLPI
SARS-CoV-2 Spike_56	SALEPLVDLPIGINI
SARS-CoV-2 Spike_57	PLVDLPIGINITRFQ
SARS-CoV-2 Spike_58	LPIGINITRFQTLLA
SARS-CoV-2 Spike_59	INITRFQTLLALHRS
SARS-CoV-2 Spike_60	RFQTLLALHRSYLTP
SARS-CoV-2 Spike_61	LLALHRSYLTPGDSS
SARS-CoV-2 Spike_62	HRSYLTPGDSSSGWT
SARS-CoV-2 Spike_63	LTPGDSSSGWTAGAA
SARS-CoV-2 Spike_64	DSSSGWTAGAAAYYV
SARS-CoV-2 Spike_65	GWTAGAAAYYVGYLQ
SARS-CoV-2 Spike_66	GAAAYYVGYLQPRTF
SARS-CoV-2 Spike_67	YYVGYLQPRTFLLKY
SARS-CoV-2 Spike_68	YLQPRTFLLKYNENG
SARS-CoV-2 Spike_69	RTFLLKYNENGTITD
SARS-CoV-2 Spike_70	LKYNENGTITDAVDC
SARS-CoV-2 Spike_71	ENGTITDAVDCALDP
SARS-CoV-2 Spike_72	ITDAVDCALDPLSET
SARS-CoV-2 Spike_73	VDCALDPLSETKCTL
SARS-CoV-2 Spike_74	LDPLSETKCTLKSFT
SARS-CoV-2 Spike_75	SETKCTLKSFTVEKG
SARS-CoV-2 Spike_76	CTLKSFTVEKGIYQT
SARS-CoV-2 Spike_77	SFTVEKGIYQTSNFR
SARS-CoV-2 Spike_78	EKGIYQTSNFRVQPT
SARS-CoV-2 Spike_79	YQTSNFRVQPTESIV
SARS-CoV-2 Spike_80	NFRVQPTESIVRFPN
SARS-CoV-2 Spike_81	QPTESIVRFPNITNL
SARS-CoV-2 Spike_82	SIVRFPNITNLCPFG
SARS-CoV-2 Spike_83	FPNITNLCPFGEVFN
SARS-CoV-2 Spike_84	TNLCPFGEVFNATRF
SARS-CoV-2 Spike_85	PFGEVFNATRFASVY
SARS-CoV-2 Spike_86	VFNATRFASVYAWNR
SARS-CoV-2 Spike_87	TRFASVYAWNRKRIS
SARS-CoV-2 Spike_88	SVYAWNRKRISNCVA
SARS-CoV-2 Spike_89	WNRKRISNCVADYSV
SARS-CoV-2 Spike_90	RISNCVADYSVLYNS
SARS-CoV-2 Spike_91	CVADYSVLYNSASFS
SARS-CoV-2 Spike_92	YSVLYNSASFSTFKC
SARS-CoV-2 Spike_93	YNSASFSTFKCYGVS
SARS-CoV-2 Spike_94	SFSTFKCYGVSPTKL
SARS-CoV-2 Spike_95	FKCYGVSPTKLNDLC
SARS-CoV-2 Spike_96	GVSPTKLNDLCFTNV
SARS-CoV-2 Spike_97	TKLNDLCFTNVYADS
SARS-CoV-2 Spike_98	DLCFTNVYADSFVIR
SARS-CoV-2 Spike_99	TNVYADSFVIRGDEV
SARS-CoV-2 Spike_100	ADSFVIRGDEVRQIA
SARS-CoV-2 Spike_101	VIRGDEVRQIAPGQT
SARS-CoV-2 Spike_102	DEVRQIAPGQTGKIA
SARS-CoV-2 Spike_103	QIAPGQTGKIADYNY
SARS-CoV-2 Spike_104	GQTGKIADYNYKLPD
SARS-CoV-2 Spike_105	KIADYNYKLPDDFTG
SARS-CoV-2 Spike_106	YNYKLPDDFTGCVIA
SARS-CoV-2 Spike_107	LPDDFTGCVIAWNSN
SARS-CoV-2 Spike_108	FTGCVIAWNSNNLDS
SARS-CoV-2 Spike_109	VIAWNSNNLDSKVGG
SARS-CoV-2 Spike_110	NSNNLDSKVGGNYNY
SARS-CoV-2 Spike_111	LDSKVGGNYNYLYRL
SARS-CoV-2 Spike_112	VGGNYNYLYRLFRKS
SARS-CoV-2 Spike_113	YNYLYRLFRKSNLKP
SARS-CoV-2 Spike_114	YRLFRKSNLKPFERD
SARS-CoV-2 Spike_115	RKSNLKPFERDISTE
SARS-CoV-2 Spike_116	LKPFERDISTEIYQA
SARS-CoV-2 Spike_117	ERDISTEIYQAGSTP
SARS-CoV-2 Spike_118	STEIYQAGSTPCNGV
SARS-CoV-2 Spike_119	YQAGSTPCNGVEGFN
SARS-CoV-2 Spike_120	STPCNGVEGFNCYFP
SARS-CoV-2 Spike_121	NGVEGFNCYFPLQSY
SARS-CoV-2 Spike_122	GFNCYFPLQSYGFQP
SARS-CoV-2 Spike_123	YFPLQSYGFQPTNGV
SARS-CoV-2 Spike_124	QSYGFQPTNGVGYQP
SARS-CoV-2 Spike_125	FQPTNGVGYQPYRVV
SARS-CoV-2 Spike_126	NGVGYQPYRVVVLSF
SARS-CoV-2 Spike_127	YQPYRVVVLSFELLH
SARS-CoV-2 Spike_128	RVVVLSFELLHAPAT
SARS-CoV-2 Spike_129	LSFELLHAPATVCGP
SARS-CoV-2 Spike_130	LLHAPATVCGPKKST
SARS-CoV-2 Spike_131	PATVCGPKKSTNLVK
SARS-CoV-2 Spike_132	CGPKKSTNLVKNKCV
SARS-CoV-2 Spike_133	KSTNLVKNKCVNFNF
SARS-CoV-2 Spike_134	LVKNKCVNFNFNGLT
SARS-CoV-2 Spike_135	KCVNFNFNGLTGTGV
SARS-CoV-2 Spike_136	FNFNGLTGTGVLTES
SARS-CoV-2 Spike_137	GLTGTGVLTESNKKF
SARS-CoV-2 Spike_138	TGVLTESNKKFLPFQ
SARS-CoV-2 Spike_139	TESNKKFLPFQQFGR
SARS-CoV-2 Spike_140	KKFLPFQQFGRDIAD
SARS-CoV-2 Spike_141	PFQQFGRDIADTTDA
SARS-CoV-2 Spike_142	FGRDIADTTDAVRDP
SARS-CoV-2 Spike_143	IADTTDAVRDPQTLE
SARS-CoV-2 Spike_144	TDAVRDPQTLEILDI
SARS-CoV-2 Spike_145	RDPQTLEILDITPCS
SARS-CoV-2 Spike_146	TLEILDITPCSFGGV
SARS-CoV-2 Spike_147	LDITPCSFGGVSVIT
SARS-CoV-2 Spike_148	PCSFGGVSVITPGTN
SARS-CoV-2 Spike_149	GGVSVITPGTNTSNQ
SARS-CoV-2 Spike_150	VITPGTNTSNQVAVL
SARS-CoV-2 Spike_151	GTNTSNQVAVLYQDV
SARS-CoV-2 Spike_152	SNQVAVLYQDVNCTE
SARS-CoV-2 Spike_153	AVLYQDVNCTEVPVA

SARS-CoV-2 Spike_154	QDVNCTEVPVAIHAD
SARS-CoV-2 Spike_155	CTEVPVAIHADQLTP
SARS-CoV-2 Spike_156	PVAIHADQLTPTWRV
SARS-CoV-2 Spike_157	HADQLTPTWRVYSTG
SARS-CoV-2 Spike_158	LTPTWRVYSTGSNVF

Subpool 2

SARS-CoV-2 Spike_159	WRVYSTGSNVFQTRA
SARS-CoV-2 Spike_160	STGSNVFQTRAGCLI
SARS-CoV-2 Spike_161	NVFQTRAGCLIGAEH
SARS-CoV-2 Spike_162	TRAGCLIGAEHVNNS
SARS-CoV-2 Spike_163	CLIGAEHVNNSYECD
SARS-CoV-2 Spike_164	AEHVNNSYECDIPIG
SARS-CoV-2 Spike_165	NNSYECDIPIGAGIC
SARS-CoV-2 Spike_166	ECDIPIGAGICASYQ
SARS-CoV-2 Spike_167	PIGAGICASYQTQTN
SARS-CoV-2 Spike_168	GICASYQTQTNSPRR
SARS-CoV-2 Spike_169	SYQTQTNSPRRARSV
SARS-CoV-2 Spike_170	QTNSPRRARSVASQS
SARS-CoV-2 Spike_171	PRRARSVASQSIIAY
SARS-CoV-2 Spike_172	RSVASQSIIAYTMSL
SARS-CoV-2 Spike_173	SQSIIAYTMSLGAEN
SARS-CoV-2 Spike_174	IAYTMSLGAENSVAY
SARS-CoV-2 Spike_175	MSLGAENSVAYSNNS
SARS-CoV-2 Spike_176	AENSVAYSNNSIAIP
SARS-CoV-2 Spike_177	VAYSNNSIAIPTNFT
SARS-CoV-2 Spike_178	NNSIAIPTNFTISVT
SARS-CoV-2 Spike_179	AIPTNFTISVTTEIL
SARS-CoV-2 Spike_180	NFTISVTTEILPVSM
SARS-CoV-2 Spike_181	SVTTEILPVSMTKTS
SARS-CoV-2 Spike_182	EILPVSMTKTSVDCT
SARS-CoV-2 Spike_183	VSMTKTSVDCTMYIC
SARS-CoV-2 Spike_184	KTSVDCTMYICGDST
SARS-CoV-2 Spike_185	DCTMYICGDSTECSN
SARS-CoV-2 Spike_186	YICGDSTECSNLLLQ
SARS-CoV-2 Spike_187	DSTECSNLLLQYGSF
SARS-CoV-2 Spike_188	CSNLLLQYGSFCTQL
SARS-CoV-2 Spike_189	LLQYGSFCTQLNRAL
SARS-CoV-2 Spike_190	GSFCTQLNRALTGIA
SARS-CoV-2 Spike_191	TQLNRALTGIAVEQD
SARS-CoV-2 Spike_192	RALTGIAVEQDKNTQ
SARS-CoV-2 Spike_193	GIAVEQDKNTQEVFA
SARS-CoV-2 Spike_194	EQDKNTQEVFAQVKQ
SARS-CoV-2 Spike_195	NTQEVFAQVKQIYKT
SARS-CoV-2 Spike_196	VFAQVKQIYKTPPIK
SARS-CoV-2 Spike_197	VKQIYKTPPIKDFGG
SARS-CoV-2 Spike_198	YKTPPIKDFGGFNFS
SARS-CoV-2 Spike_199	PIKDFGGFNFSQILP
SARS-CoV-2 Spike_200	FGGFNFSQILPDPSK
SARS-CoV-2 Spike_201	NFSQILPDPSKPSKR
SARS-CoV-2 Spike_202	ILPDPSKPSKRSFIE
SARS-CoV-2 Spike_203	PSKPSKRSFIEDLLF
SARS-CoV-2 Spike_204	SKRSFIEDLLFNKVT
SARS-CoV-2 Spike_205	FIEDLLFNKVTLADA
SARS-CoV-2 Spike_206	LLFNKVTLADAGFIK
SARS-CoV-2 Spike_207	KVTLADAGFIKQYGD
SARS-CoV-2 Spike_208	ADAGFIKQYGDCLGD
SARS-CoV-2 Spike_209	FIKQYGDCLGDIAAR
SARS-CoV-2 Spike_210	YGDCLGDIAARDLIC
SARS-CoV-2 Spike_211	LGDIAARDLICAQKF
SARS-CoV-2 Spike_212	AARDLICAQKFNGLT
SARS-CoV-2 Spike_213	LICAQKFNGLTVLPP
SARS-CoV-2 Spike_214	QKFNGLTVLPPLLTD
SARS-CoV-2 Spike_215	GLTVLPPLLTDEMIA
SARS-CoV-2 Spike_216	LPPLLTDEMIAQYTS
SARS-CoV-2 Spike_217	LTDEMIAQYTSALLA
SARS-CoV-2 Spike_218	MIAQYTSALLAGTIT
SARS-CoV-2 Spike_219	YTSALLAGTITSGWT
SARS-CoV-2 Spike_220	LLAGTITSGWTFGAG
SARS-CoV-2 Spike_221	TITSGWTFGAGAALQ
SARS-CoV-2 Spike_222	GWTFGAGAALQIPFA
SARS-CoV-2 Spike_223	GAGAALQIPFAMQMA
SARS-CoV-2 Spike_224	ALQIPFAMQMAYRFN
SARS-CoV-2 Spike_225	PFAMQMAYRFNGIGV
SARS-CoV-2 Spike_226	QMAYRFNGIGVTQNV
SARS-CoV-2 Spike_227	RFNGIGVTQNVLYEN
SARS-CoV-2 Spike_228	IGVTQNVLYENQKLI
SARS-CoV-2 Spike_229	QNVLYENQKLIANQF
SARS-CoV-2 Spike_230	YENQKLIANQFNSAI
SARS-CoV-2 Spike_231	KLIANQFNSAIGKIQ
SARS-CoV-2 Spike_232	NQFNSAIGKIQDSLS
SARS-CoV-2 Spike_233	SAIGKIQDSLSSTAS
SARS-CoV-2 Spike_234	KIQDSLSSTASALGK
SARS-CoV-2 Spike_235	SLSSTASALGKLQDV
SARS-CoV-2 Spike_236	TASALGKLQDVVNQN
SARS-CoV-2 Spike_237	LGKLQDVVNQNAQAL
SARS-CoV-2 Spike_238	QDVVNQNAQALNTLV
SARS-CoV-2 Spike_239	NQNAQALNTLVKQLS
SARS-CoV-2 Spike_240	QALNTLVKQLSSNFG
SARS-CoV-2 Spike_241	TLVKQLSSNFGAISS
SARS-CoV-2 Spike_242	QLSSNFGAISSVLND
SARS-CoV-2 Spike_243	NFGAISSVLNDILSR
SARS-CoV-2 Spike_244	ISSVLNDILSRLDKV
SARS-CoV-2 Spike_245	LNDILSRLDKVEAEV
SARS-CoV-2 Spike_246	LSRLDKVEAEVQIDR
SARS-CoV-2 Spike_247	DKVEAEVQIDRLITG
SARS-CoV-2 Spike_248	AEVQIDRLITGRLQS
SARS-CoV-2 Spike_249	IDRLITGRLQSLQTY
SARS-CoV-2 Spike_250	ITGRLQSLQTYVTQQ
SARS-CoV-2 Spike_251	LQSLQTYVTQQLIRA
SARS-CoV-2 Spike_252	QTYVTQQLIRAAEIR
SARS-CoV-2 Spike_253	TQQLIRAAEIRASAN
SARS-CoV-2 Spike_254	IRAAEIRASANLAAT
SARS-CoV-2 Spike_255	EIRASANLAATKMSE
SARS-CoV-2 Spike_256	SANLAATKMSECVLG
SARS-CoV-2 Spike_257	AATKMSECVLGQSKR
SARS-CoV-2 Spike_258	MSECVLGQSKRVDFC
SARS-CoV-2 Spike_259	VLGQSKRVDFCGKGY
SARS-CoV-2 Spike_260	SKRVDFCGKGYHLMS
SARS-CoV-2 Spike_261	DFCGKGYHLMSFPQS
SARS-CoV-2 Spike_262	KGYHLMSFPQSAPHG
SARS-CoV-2 Spike_263	LMSFPQSAPHGVVFL
SARS-CoV-2 Spike_264	PQSAPHGVVFLHVTY
SARS-CoV-2 Spike_265	PHGVVFLHVTYVPAQ
SARS-CoV-2 Spike_266	VFLHVTYVPAQEKNF
SARS-CoV-2 Spike_267	VTYVPAQEKNFTTAP
SARS-CoV-2 Spike_268	PAQEKNFTTAPAICH
SARS-CoV-2 Spike_269	KNFTTAPAICHDGKA
SARS-CoV-2 Spike_270	TAPAICHDGKAHFPR
SARS-CoV-2 Spike_271	ICHDGKAHFPREGVF
SARS-CoV-2 Spike_272	GKAHFPREGVFVSNG
SARS-CoV-2 Spike_273	FPREGVFVSNGTHWF
SARS-CoV-2 Spike_274	GVFVSNGTHWFVTQR
SARS-CoV-2 Spike_275	SNGTHWFVTQRNFYE
SARS-CoV-2 Spike_276	HWFVTQRNFYEPQII
SARS-CoV-2 Spike_277	TQRNFYEPQIITTDN
SARS-CoV-2 Spike_278	FYEPQIITTDNTFVS
SARS-CoV-2 Spike_279	QIITTDNTFVSGNCD
SARS-CoV-2 Spike_280	TDNTFVSGNCDVVIG
SARS-CoV-2 Spike_281	FVSGNCDVVIGIVNN
SARS-CoV-2 Spike_282	NCDVVIGIVNNTVYD
SARS-CoV-2 Spike_283	VIGIVNNTVYDPLQP
SARS-CoV-2 Spike_284	VNNTVYDPLQPELDS
SARS-CoV-2 Spike_285	VYDPLQPELDSFKEE
SARS-CoV-2 Spike_286	LQPELDSFKEELDKY
SARS-CoV-2 Spike_287	LDSFKEELDKYFKNH
SARS-CoV-2 Spike_288	KEELDKYFKNHTSPD
SARS-CoV-2 Spike_289	DKYFKNHTSPDVDLG
SARS-CoV-2 Spike_290	KNHTSPDVDLGDISG
SARS-CoV-2 Spike_291	SPDVDLGDISGINAS
SARS-CoV-2 Spike_292	DLGDISGINASVVNI
SARS-CoV-2 Spike_293	ISGINASVVNIQKEI
SARS-CoV-2 Spike_294	NASVVNIQKEIDRLN
SARS-CoV-2 Spike_295	VNIQKEIDRLNEVAK
SARS-CoV-2 Spike_296	KEIDRLNEVAKNLNE
SARS-CoV-2 Spike_297	RLNEVAKNLNESLID
SARS-CoV-2 Spike_298	VAKNLNESLIDLQEL
SARS-CoV-2 Spike_299	LNESLIDLQELGKYE
SARS-CoV-2 Spike_300	LIDLQELGKYEQYIK
SARS-CoV-2 Spike_301	QELGKYEQYIKWPWY
SARS-CoV-2 Spike_302	KYEQYIKWPWYIWLG
SARS-CoV-2 Spike_303	YIKWPWYIWLGFIAG
SARS-CoV-2 Spike_304	PWYIWLGFIAGLIAI
SARS-CoV-2 Spike_305	WLGFIAGLIAIVMVT
SARS-CoV-2 Spike_306	IAGLIAIVMVTIMLC
SARS-CoV-2 Spike_307	IAIVMVTIMLCCMTS
SARS-CoV-2 Spike_308	MVTIMLCCMTSCCSC
SARS-CoV-2 Spike_309	MLCCMTSCCSCLKGC
SARS-CoV-2 Spike_310	MTSCCSCLKGCCSCG
SARS-CoV-2 Spike_311	CSCLKGCCSCGSCCK
SARS-CoV-2 Spike_312	KGCCSCGSCCKFDED
SARS-CoV-2 Spike_313	SCGSCCKFDEDDSEP
SARS-CoV-2 Spike_314	CCKFDEDDSEPVLKG
SARS-CoV-2 Spike_315	DEDDSEPVLKGVKLH
SARS-CoV-2 Spike_316	SEPVLKGVKLHYT

Supporting information: Selection of amyloidogenic segments for peptide synthesis and comparison with peptides rendered by *in silico* elastase digestion

WALTZ prediction “high specificity”, pH 7.0 [1], on SARS-CoV-2 spike protein P0DTC2 indicated with asterisk under the sequence. The synthesized peptide sequences highlighted in colors corresponding to Figure S2A-B. Segments highlighted in grey are peptides predicted to be the result of cleaving with Neutrophil elastase using Expasy peptide cutter and rendering peptides with 3 or more consecutive amino acids in one of the amyloidogenic regions.

```
>sp|P0DTC2|SPIKE_SARS2
MFVFLVLLPLVSSQCVNLTTRTQLPPAYTNSFTRGVYYPDKVFRSSVLHSTQDLFLPFFS

NVTWFHAIHVSGTNGTKRFDNPVLPFNDGVYFASTEKSNIIRGWIFGTTLDSKTQSLLIV

NNATNVVIKVCEFQFCNDPFLGVYYHKNNKSWMESEFRVYSSANNCTFEYVSQPFLMDLE

GKQGNFKNLREFVFKNIDGYFKIYSKHTPINLVRDLPQGFSALEPLVDLPIGINITRFQT
                     ******
                FKNIDGYFKIYSKHTPINLV

LLALHRSYLTPGDSSSGWTAGAAAYYVGYLQPRTFLLKYNENGTITDAVDCALDPLSETK
                            *********
                                   GYLQPRTFLLKYNENGTITDA

CTLKSFTVEKGIYQTSNFRVQPTESIVRFPNITNLCPFGEVFNATRFASVYAWNRKRISN

CVADYSVLYNSASFSTFKCYGVSPTKLNDLCFTNVYADSFVIRGDEVRQIAPGQTGKIAD
    ********
       LYNSA

YNYKLPDDFTGCVIAWNSNNLDSKVGGNYNYLYRLFRKSNLKPFERDISTEIYQAGSTPC

NGVEGFNCYFPLQSYGFQPTNGVGYQPYRVVVLSFELLHAPATVCGPKKSTNLVKNKCVN
                                                        ****
                                                           N

FNFNGLTGTGVLTESNKKFLPFQQFGRDIADTTDAVRDPQTLEILDITPCSFGGVSVITP
****
FNFNGLTGTGV

GTNTSNQVAVLYQDVNCTEVPVAIHADQLTPTWRVYSTGSNVFQTRAGCLIGAEHVNNSY
       ******
          LYQDV


ECDIPIGAGICASYQTQTNSPRRARSVASQSIIAYTMSLGAENSVAYSNNSIAIPTNFTI
                            *********
                            SQSIIA
SVTTEILPVSMTKTSVDCTMYICGDSTECSNLLLQYGSFCTQLNRALTGIAVEQDKNTQE

VFAQVKQIYKTPPIKDFGGFNFSQILPDPSKPSKRSFIEDLLFNKVTLADAGFIKQYGDC

LGDIAARDLICAQKFNGLTVLPPLLTDEMIAQYTSALLAGTITSGWTFGAGAALQIPFAM

QMAYRFNGIGVTQNVLYENQKLIANQFNSAIGKIQDSLSSTASALGKLQDVVNQNAQALN

TLVKQLSSNFGAISSVLNDILSRLDKVEAEVQIDRLITGRLQSLQTYVTQQLIRAAEIRA

SANLAATKMSECVLGQSKRVDFCGKGYHLMSFPQSAPHGVVFLHVTYVPAQEKNFTTAPA

ICHDGKAHFPREGVFVSNGTHWFVTQRNFYEPQIITTDNTFVSGNCDVVIGIVNNTVYDP

LQPELDSFKEELDKYFKNHTSPDVDLGDISGINASVVNIQKEIDRLNEVAKNLNESLIDL
                                  *****
QELGKYEQYIKWPWYIWLGFIAGLIAIVMVTIMLCCMTSCCSCLKGCCSCGSCCKFDEDD

SEPVLKGVKLHYT
```

Figure S1: Fibrils formed from bulk S-protein peptide library

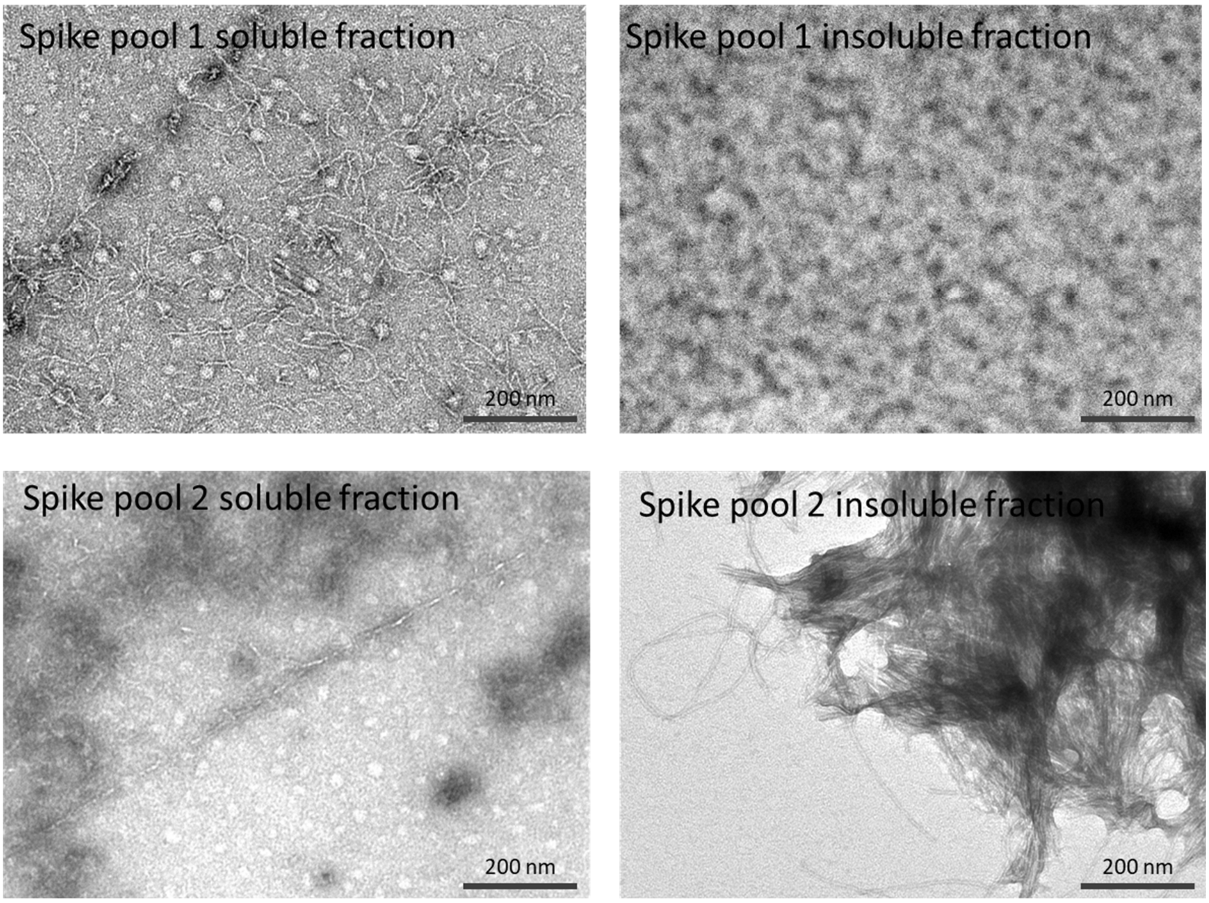

Figure S1. Negative stain TEM micrographs of **A.** Long single filament fibrils formed from PBS soluble fraction S-protein peptide library pool 1. **B.** Amorphous aggregates from PBS insoluble fraction S-protein peptide library pool 1. **C.** Short fibrils and amorphous aggregates formed from PBS soluble fraction S-protein peptide library pool 2 **B.** Heavily clustered long fibrils from PBS insoluble fraction S-protein peptide library pool 2.

Figure S2: Structural context of the Spike peptides

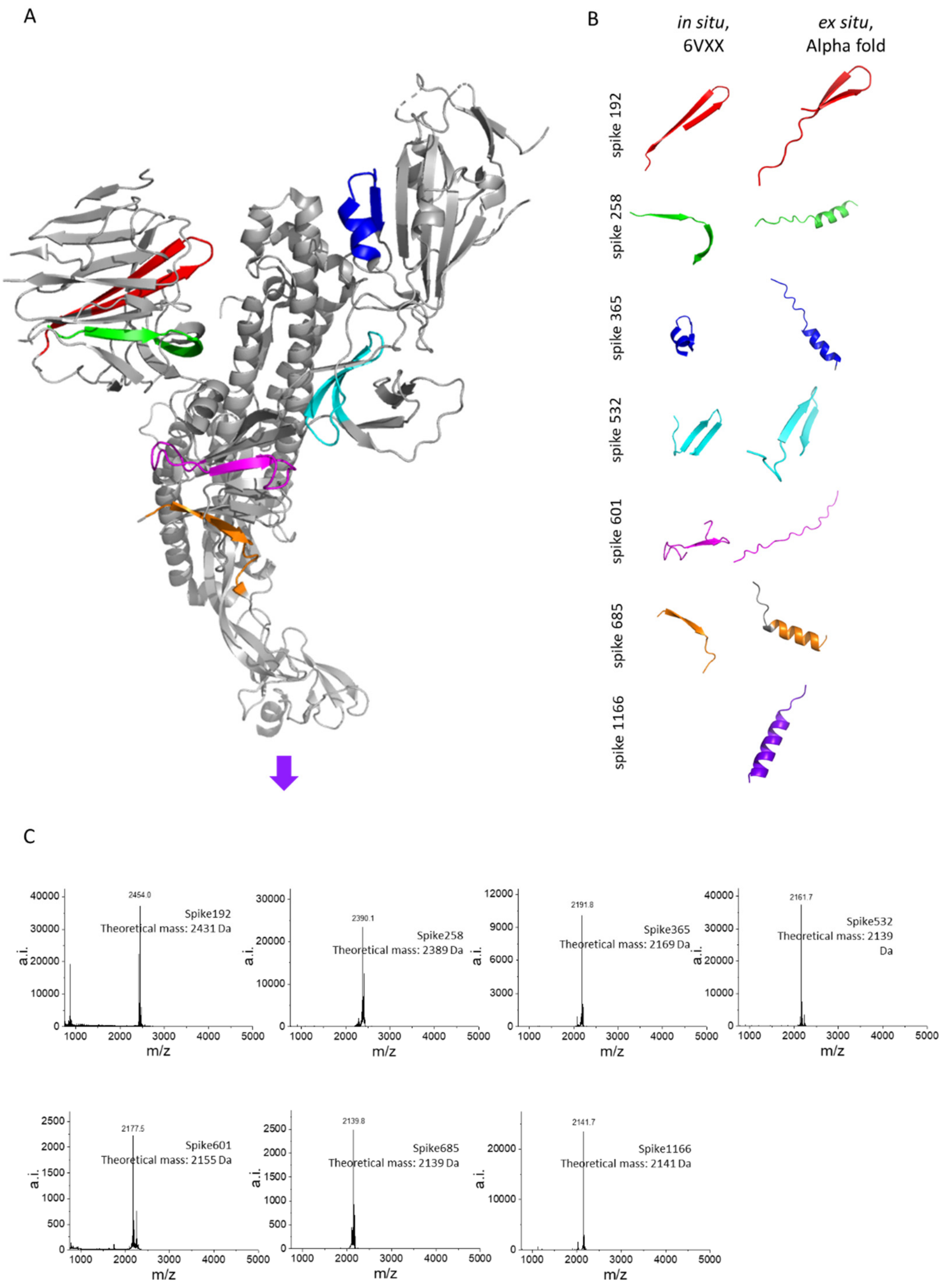

Figure S2: A. The structure of one protomer of the trimeric SARS-CoV-2 S-protein in its closed state, PDB code: 6VXX [4] with the predicted full sequence of the amyloidogenic peptides highlighted in the same colors as the predictions in main text Table 1, Figure 3 and Supporting information. **B**. Conformation of peptides within the folded S-protein in comparison with AlphaFold 2 models of the synthetic peptides (sequences as in Table 1). **C**. MALDI-ToF spectra of synthetic peptides dissolved in PBS buffer (10% HFIP) at 0.1 mg/ml to verify sequence and purity. Spike192, Spike365, Spike532, Spike601 show +22 Da due to Na^+ complex formation.

Figure S3: Fibrillation of mix of the seven Spike peptides

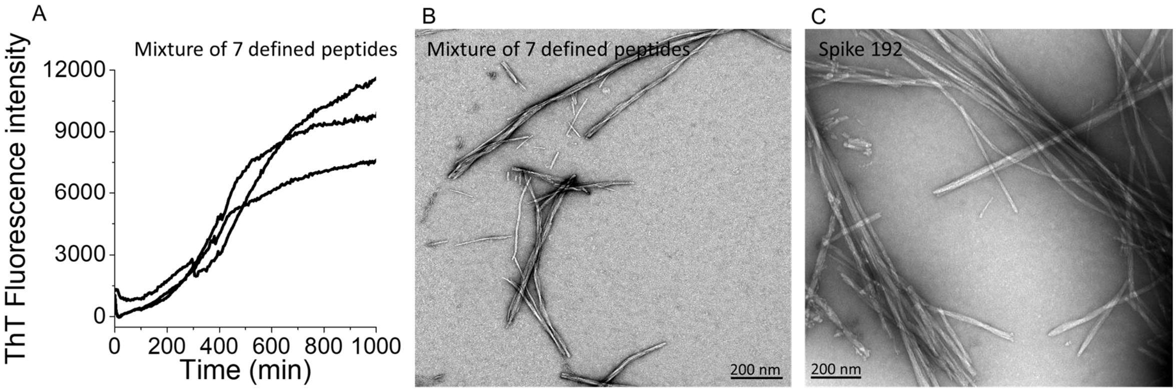

Figure S3. Amyloid fibrillation of 7 mixed SARS-CoV-2 S-peptides (total concentration 0.1 mg/ml). **A.** Fibril formation kinetics monitored by ThT fluorescence. **B.** Fibrillar structures by negative stain TEM. **C.** Fibrillar structures by negative stain TEM of Spike192 resembling the mix

Figure S4: Thermal denaturation by DSF

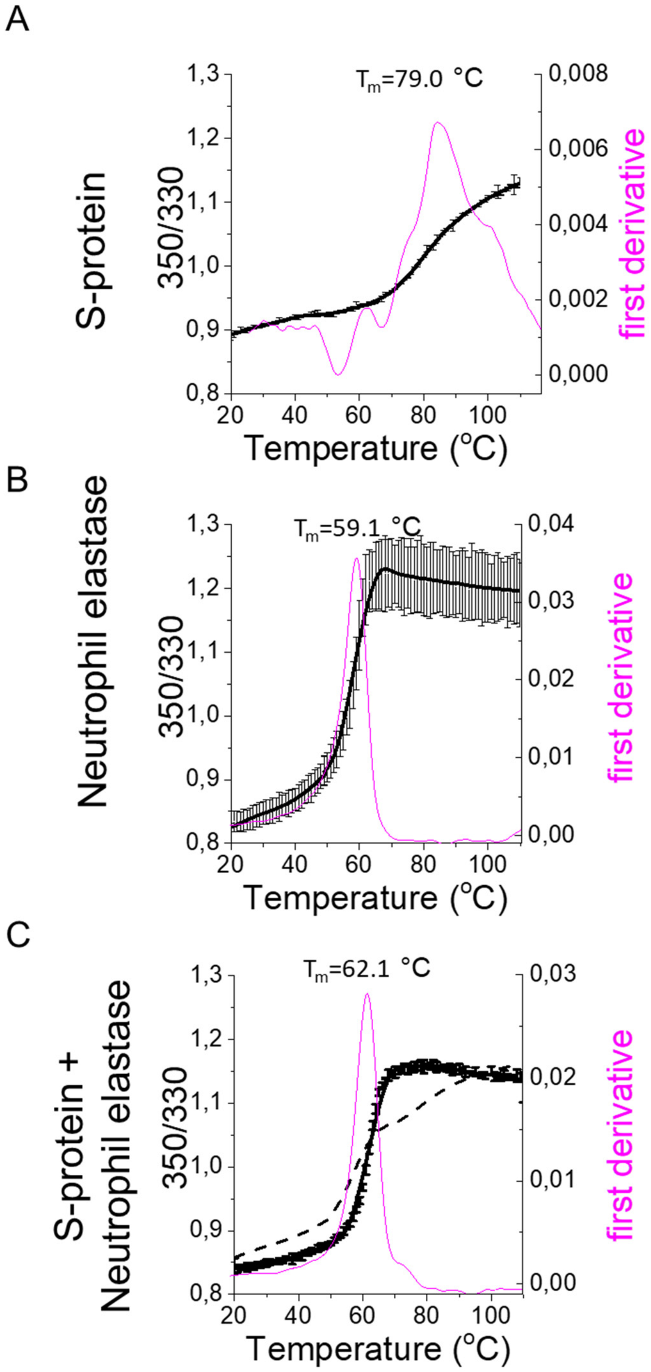

Figure S4: Thermal denaturation by differential scanning fluorimetry (DSF) at pH 8.4 of **A**) S-protein **B**) Neutrophil elastase **C**) co-incubation of S-protein and Neutrophil elastase. Black curves show increase in tryptophan exposure to polar environment by emission ratio 350nm/330nm. Data is the average of two separate experiments with standard deviations. The dashed line in (**C**) represents the summed signals from A and B which does not fit the experimental data of the co-incubated proteins indicating S-protein cleavage by elastase. Magenta curve shows the first derivative of the thermal denaturation curve, accentuating the infliction points during denaturation. S-protein (A) in particular, displays a complex denaturation transition, dictated by the dissociation of the trimer as well as unfolding of the different subunits within each monomer.

Figure S5: Raw transmission electron micrographs of full-length SARS-CoV-2 S-protein, Neutrophil Elastase and S-protein + Neutrophil Elastase incubated at 37 °C for 24h

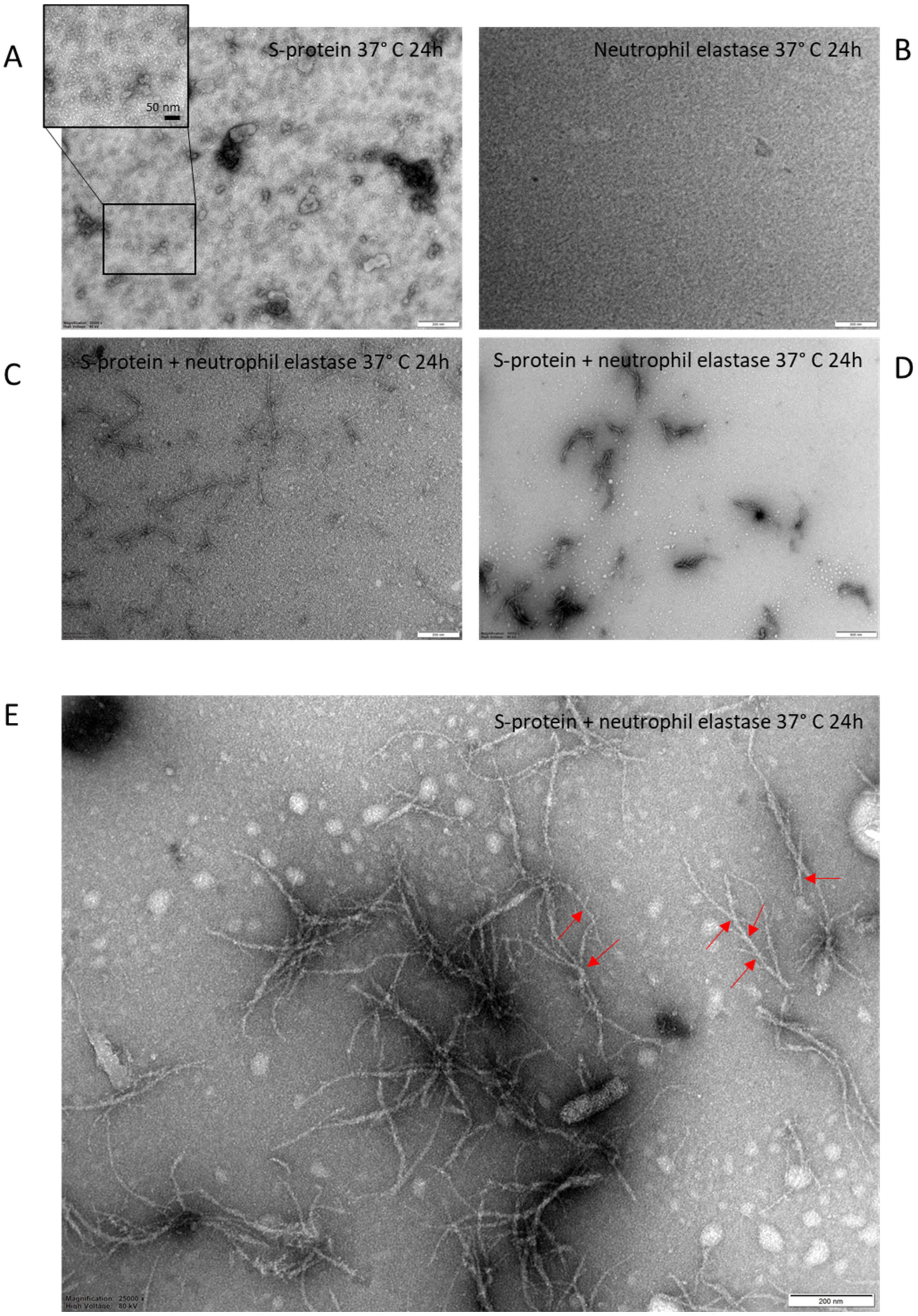

Figure S5. Negative stain TEM micrographs of **A.** S-protein, inset highlights the trimeric spike protein as displayed in [5] **B.** neutrophil elastase and **C-E**. S-Protein + elastase co-incubated in vitro at 37 °C for 24 h. S-protein trimer structures are visible in **A.** Fibrils were only formed in the co-incubated samples showing single filament fibrils in **C** and clusters of amyloid-like fibrils in **D.** The unusual fibril morphology with evident branching is clearly visible in the high magnification image in **E** and are highlighted with red arrows.

Figure S6: *In silico* mutation of one amino acid in the amyloidogenic segment in Spike192

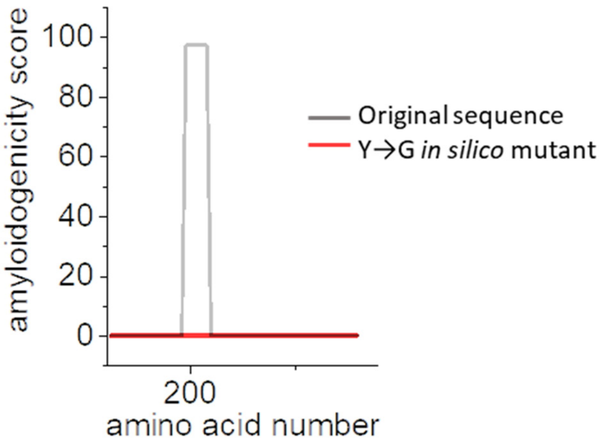


```
GKQGNFKNLREFVFKNIDGYFKIYSKHTPINLVRDLPQGFSALEPLVDLPIGINITRFQT
                  ******
GKQGNFKNLREFVFKNIDGYFKIGSKHTPINLVRDLPQGFSALEPLVDLPIGINITRFQT
```

Figure S6: *In silico* replacement of the final tyrosine residue in the amino acid stretch surrounding Spike192 peptide to a glycine abolished the predicted amyloidogenicity of the peptide.

Figure S7: Fluorescence micrographs and spectra of Spike192 fibrils stained with fluorescent analogs of amyloid PET tracers

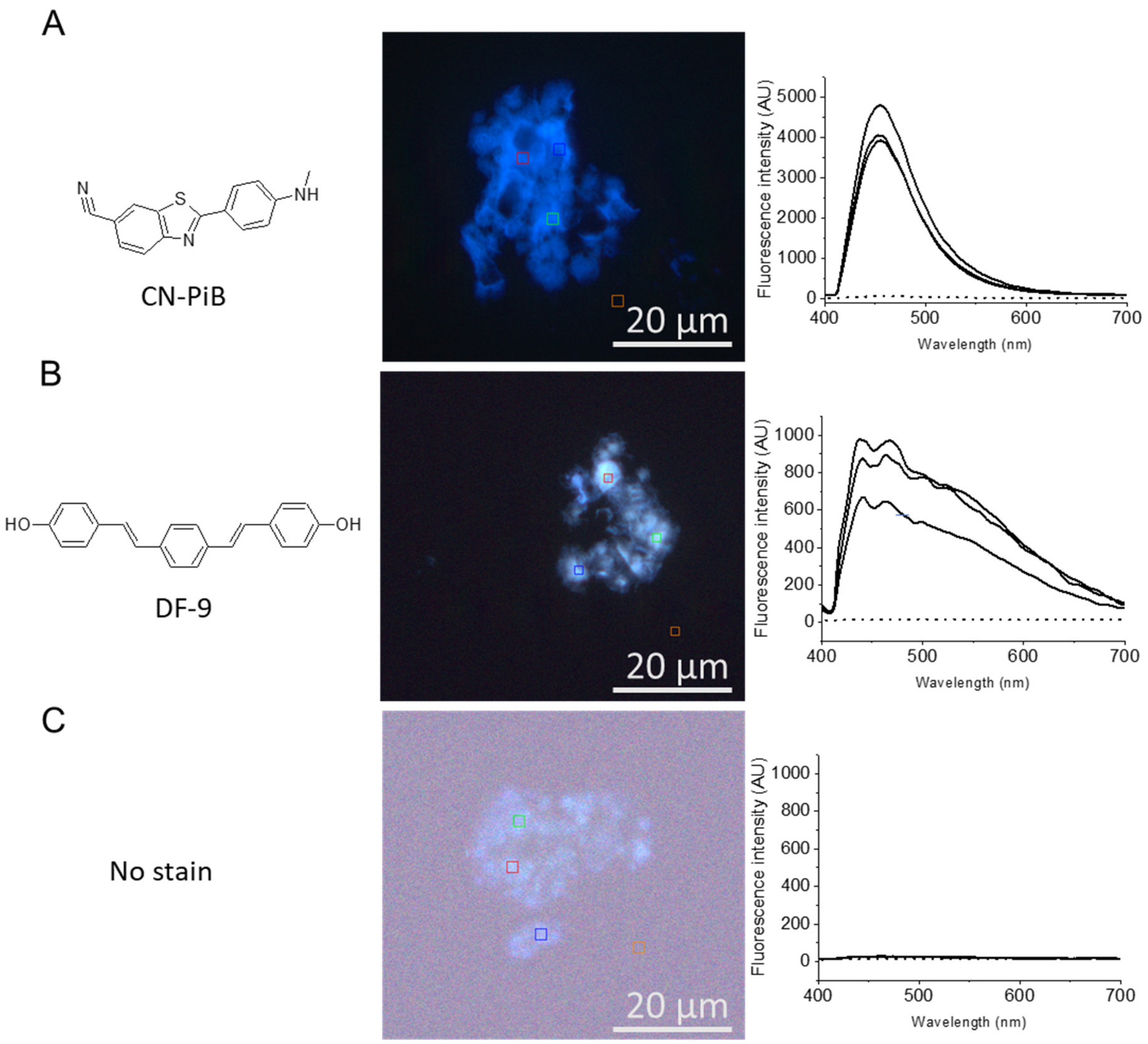

Figure S7. Fibrils of Spike192 stained with ligands CN-PiB (**A**) and DF-9 (**B**), fluorescent analogues of the PET tracers ^{11}C-PiB and ^{18}F-Florbetaben respectively, imaged with fluorescence hyperspectral microscopy. Both ligands show intense staining of fibrils with the expected emission spectra bound to amyloid fibrils. Spectra from three regions of interest (ROI) are depicted to the right of the micrographs, marked with colored squares in the micrographs. The brown colored ROIs are background signals outside of the aggregates and are as expected at very low intensity in the spectral graphs (dotted line). **C.** Autofluorescence from Spike192 fibrils at identical microscope settings shown in C is negligible as evident from the poor image contrast and intensity of the spectra.

Figure S8: WALTZ amyloidogenic sequence predictions of S-protein sequences from seven corona viruses known to infect humans

A

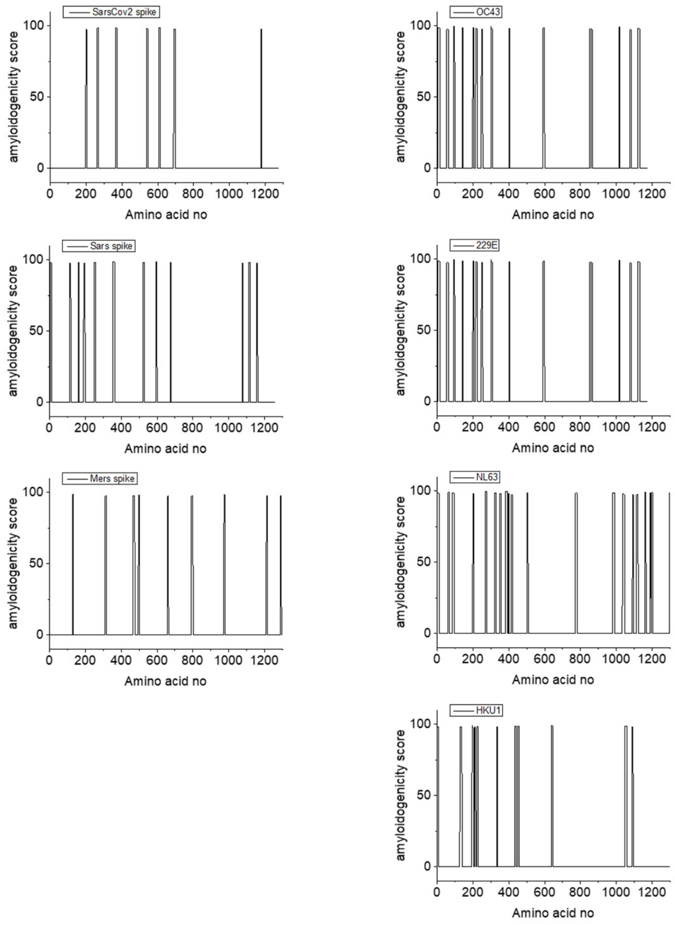

B

```
MERS                EDEILEWFGITQTAQGVHLFSSRYVD----LYGG--------------N-MFQFATLPVYD
SARS                SLDVSEKSG---NFKHLREFVFKNKDGFLYVYKGYQPIDVVRDLPSGFNTLKPIFKLPLGI
SARSCOV2            LMDLEGKQG---NFKNLREFVFKNIDGYFKIYSKHTPINLVRDLPQGFSALEPLVDLPIGI
                      ::    *   . : :: *  :  *    :*                . :  :  **:
                  176                                                           233

OC43                TYDVNAT------YLYFHFYQEGGTFYAYFTDTG------FVTKF-----LFNVYLGM
229E                NGTNTSH------SVCNGCVGHSENVFAVESGGY------IPSNF-----AFNNWFLL
NL63                NGRIVNY------TVCDDCNGYTDNIFSVQQDGR------IPNGF-----SFNNWFLL
HKU1                FTYNVSTD------WLYFFYQERGTFYAYYADSG------MPTTF-----LFSLYLGT
SARSCOV2            LMDLEGKQGNFKNLREFVFKNIDGYFKIYSKHTPINLVRDLPQGFSALEPLVDLPIGI
                                             .              :   *      ..  :
                  176                                                        233
```

Figure S8 A) WALTZ prediction of amyloidogenic sequences in Spike proteins from several corona viruses known to infect humans **B)** Sequence alignments of spike192 and flanking amino acids from the three corona viruses causing severe disease. MERS S-protein does not contain the amyloidogenic sequence, SARS S-protein contains a similar amyloidogenic sequence as SARS-CoV-2 S-protein in this part of the amino acid sequence. SARS-CoV-2 S-protein segment 194-213, highlighted in red was a peptide predicted for elastase cleavage.

References

1. Maurer-Stroh, S., et al., *Exploring the sequence determinants of amyloid structure using position-specific scoring matrices.* Nat Methods, 2010. **7**(3): p. 237-42.
2. Jumper, J., et al., *Highly accurate protein structure prediction with AlphaFold.* Nature, 2021. **596**(7873): p. 583-589.
3. Terasawa, F., et al., *In vitro fibrin clot formation and fibrinolysis using heterozygous plasma fibrinogen from gammaAsn319, Asp320 deletion dysfibrinogen, Otsu I.* Thromb Res, 2006. **118**(5): p. 651-61.
4. Walls, A.C., et al., *Structure, Function, and Antigenicity of the SARS-CoV-2 Spike Glycoprotein.* Cell, 2020. **181**(2): p. 281-292 e6.
5. Edwards, R.J., et al., *Cold sensitivity of the SARS-CoV-2 spike ectodomain.* Nat Struct Mol Biol, 2021. **28**(2): p. 128-131.
